# Integrating transcriptomic, physiological, and biochemical studies revealing the role of endogenous ABA and GA_3_ in the germination of quinoa seed

**DOI:** 10.3389/fpls.2026.1722072

**Published:** 2026-03-12

**Authors:** Xueying Li, Xiuzhang Wang, Ailin Li, Ou Luo, Yanxia Sun, Xiaoyong Wu

**Affiliations:** Key Laboratory of Coarse Cereal Processing, Ministry of Agriculture and Rural Affairs, Sichuan Engineering and Technology Research Center of Coarse Cereal Industrialization, School of Food and Biological Engineering, Chengdu University, Chengdu, Sichuan, China

**Keywords:** abscisic acid (ABA), gibberellic acid (GA), quinoa, seed germination, transcriptome

## Abstract

**Background:**

Plant endogenous hormones play crucial roles in seed germination. Among them, abscisic acid (ABA) and gibberellin (GA), two antagonistic hormones, are central regulators. However, their precise mechanisms in quinoa seed germination remain incompletely understood.

**Methods:**

Thus, by combining physiological and transcriptome analyses, this study provides insights into the ABA/GA3-mediated regulatory mechanisms during seed germination in quinoa. employs quinoa seed germination as a model to simulate PHS, with a primary focus on analyzing the alterations in starch, protein, soluble sugar, and endogenous ABA and GA_3_ content in quinoa seeds pre- and post-germination. Additionally, the study investigates the enzymatic activities associated with these two hormones. Also, the transcriptome data analysis before and after seed germination elucidate the mechanisms by which endogenous ABA and GA_3_ regulate quinoa seed germination.

**Results and discussion:**

The germination leads to an increase in the concentrations of soluble sugars, proteins, maltose, and glucose. Quinoa seeds exhibit insensitivity to ABA, while GA_3_ plays a significant role in promoting seed germination. Transcriptome revealed upregulation of starch and sucrose metabolism and the EMP pathway and TCA cycle were enhanced during seed germination. Fifteen crucial genes related to ABA, GA_3_, starch/sucrose metabolism, and EMP pathway in quinoa germination were identified. Notably, unlike most crops, the elevated endogenous ABA levels are inadequate to impede the germination of quinoa seeds or quinoa seeds exhibit insensitivity to ABA. The analysis of transcriptome data demonstrated an upregulation of the starch and sucrose metabolism pathways, as well as glycolysis and the tricarboxylic acid cycle, during the germination process of quinoa seeds. These findings provide a foundational theoretical framework for elucidating the intrinsic mechanisms underlying quinoa germination and preharvest sprouting.

## 1 Introduction

Quinoa (*Chenopodium quinoa* Wild.) is a highly nutritious grain, containing significant amounts of protein, vitamins, minerals, dietary fiber, plant sterols, and phenolic compounds, while also being gluten-free. Additionally, it provides essential amino acids that meeting the human dietary needs. Recognized for its nutritional richness, it is commonly referred to as a “super grain” or “golden grain” (Pathan and Siddiqui, 2022; Filho et al., 2017; Tabatabaei et al., 2022). Due to its rich nutritional value, it has been referred to as the perfect and strategic food by Food and Agriculture Organization of the United Nations (FAO) and can potentially become a substitute for animal protein (Dakhili et al., 2019). Due to the absence of dormancy in quinoa seeds, quinoa is prone to pre-harvest sprouting (PHS) (López-Cristoffanini et al., 2019; Ceccato et al., 2011).

PHS is defined as the occurrence of sprouting in grains while still on the plant, triggered by favorable germination conditions prior to harvest (Nakamura, 2018). PHS results in the degradation of proteins, starch, fats, and other essential nutrients in grains, leading to a significant decrease in their nutritional and economic worth (Vetch et al., 2019). Global data indicates that PHS annually causes economic losses amounting to one billion US dollars (Tai et al., 2021). This phenomenon is intricately linked to seed dormancy and the germination process. Seed germination is a vital stage in the physiological development of crops, playing a critical role in determining quality and yield. It involves a series of orderly physiological and morphological changes that occur following the absorption of water and expansion of seeds (Guardianelli et al., 2022). The influencing factors can be categorized into two main groups: external factors such as light, temperature, and water, and internal factors including spike shape, hormones, carbohydrate metabolism, proteinase, reactive oxygen species, seed maturity, and crop variety (Rabieyan et al., 2022; Tai et al., 2021; Barrero et al., 2020; Javaid et al., 2022; Klupczyńska and Pawłowski, 2021; Ali et al., 2022; Li et al., 2022). Seed germination is dependent on the regulation of plant hormones abscisic acid (ABA) and gibberellic acid (GAs), as well as the utilization of energy reserves such as starch and soluble sugars.

Research findings have demonstrated the significant roles of plant hormones ABA and GAs in the regulation of seed germination and plant maturation (Li et al., 2022; Barreto et al., 2020; Finkelstein, 2013). The sesquiterpene compound ABA is derived from its precursor, isopentenyl pyrophosphate (IPP), through synthesis in the carotenoid pathway. This process involves a series of enzymes, including Phytoene synthase (PSY), β-carotene hydroxylase (BCH), Zeaxanthin epoxidase (ZEP), Violaxanthin deepoxidase (VDE), 9-cis-epoxycarotenoid dioxygenase (NCED), Short-chain dehydrogenase/Reductase (SDR), and Aldehyde oxidases (AAO) (Wang et al., 2021). The degradation of ABA is mediated by hydroxylation reactions catalyzed by P450 cytochrome monooxygenases from the CYP707A family, resulting in a reduction of ABA levels. Specifically, the enzyme responsible for this process is referred to as ABA 8’-hydroxylase (ABA8’-H) (Ali et al., 2022; Yoshida et al., 2019).

In analogy to ABA, the biosynthetic pathway of GAs is also a complex process. Currently, 136 forms of GAs have been identified and classified into C20 and C19 types based on carbon atom count (Wuddineh et al., 2015). Among these forms, only GA_1_, GA_3_, GA_4_, and GA_7_ have demonstrated biological activity (Binenbaum et al., 2018). The precursor for GAs synthesis is Geranylgeranyl diphosphate (GGPP), and GA12 is synthesized through the enzymatic reactions catalyzed by Copalyl diphosphate synthesis (CPS), ent-Kaurene synthesis (KS), ent-Kaurene oxidase (KO), and ent-Kaurenoic acid oxidase (KAO). GA20 oxidase (GA20ox) catalyzes the conversion of GA12 into GA9 and G20, followed by the synthesis of four bioactive GA types, which is catalyzed by GA3 oxidase (GA3ox) (López-Cristoffanini et al., 2019; Yamaguchi, 2008; Urbanová et al., 2013; Binenbaum et al., 2018; Lor and Olszewski, 2015; Tuan et al., 2018).The deactivation of GAs involves the isomerization of -OH positions in GAs, catalyzed by GA2 oxidase (GA2ox), which plays a crucial role in regulating GAs catabolism (Hedden, 2001).

Except for hormones, notable alterations in the content and activity of storage compounds such as starch, protease, amylase, amino acids, and soluble sugars also take place during seed germination (Damaris et al., 2019; Liu et al., 2018; Zhang et al., 2023; Bakhshy et al., 2020; Hajihashemi et al., 2020; Guardianelli et al., 2022). Studies in celery (Li et al., 2022), soybeans (Huang et al., 2022), and mustard (Wei et al., 2023) have demonstrated that significant changes occur in sugar substances during seed germination, leading to enhanced energy metabolism. The utilization of starch and soluble sugars is essential as the primary energy source for seed germination (Liu et al., 2021).

Currently, there is a scarcity of research that incorporates indicators such as ABA and GAs content, sugar content alterations, and molecular mechanisms in the study of quinoa germination. Transcriptome sequencing includes short-read sequencing, long-read RNA sequencing, and direct RNA sequencing. Illumina, as the primary platform for short-read sequencing, is also the most widely used and mature sequencing platform (Stark et al., 2019). Currently, researchers have used RNA sequencing (RNA-seq) technology to learn gene expression changes during seed germination and identify key germination genes in hazelnut (Liu et al., 2020), wheat (Yu et al., 2014), and rice (Zhang et al., 2023).

In this study, Cheng Li (CL-2) was utilized as the material to investigate the mechanism of endogenous physiological and biochemical indicators, as well as gene expression during seed germination. The content of starch, soluble sugar, soluble protein, amylase, maltose, sucrose, fructose, glucose, ABA, GA_3_, and the related enzyme activities involved in ABA and GA_3_ synthesis metabolism were quantified. Additionally, transcriptome data obtained at 4-hour and 12-hour time points after germination were analyzed to examine the dynamic changes in various indicators during germination. Moreover, the transcriptome was utilized to evaluate the expression of functionally related genes during the germination of quinoa. This study aimed to elucidate the roles of various markers in the seed germination process and offer valuable insights for further research on quinoa germination.

## 2 Material and methods

### 2.1 Preparation of experimental materials

**Figure 1:**
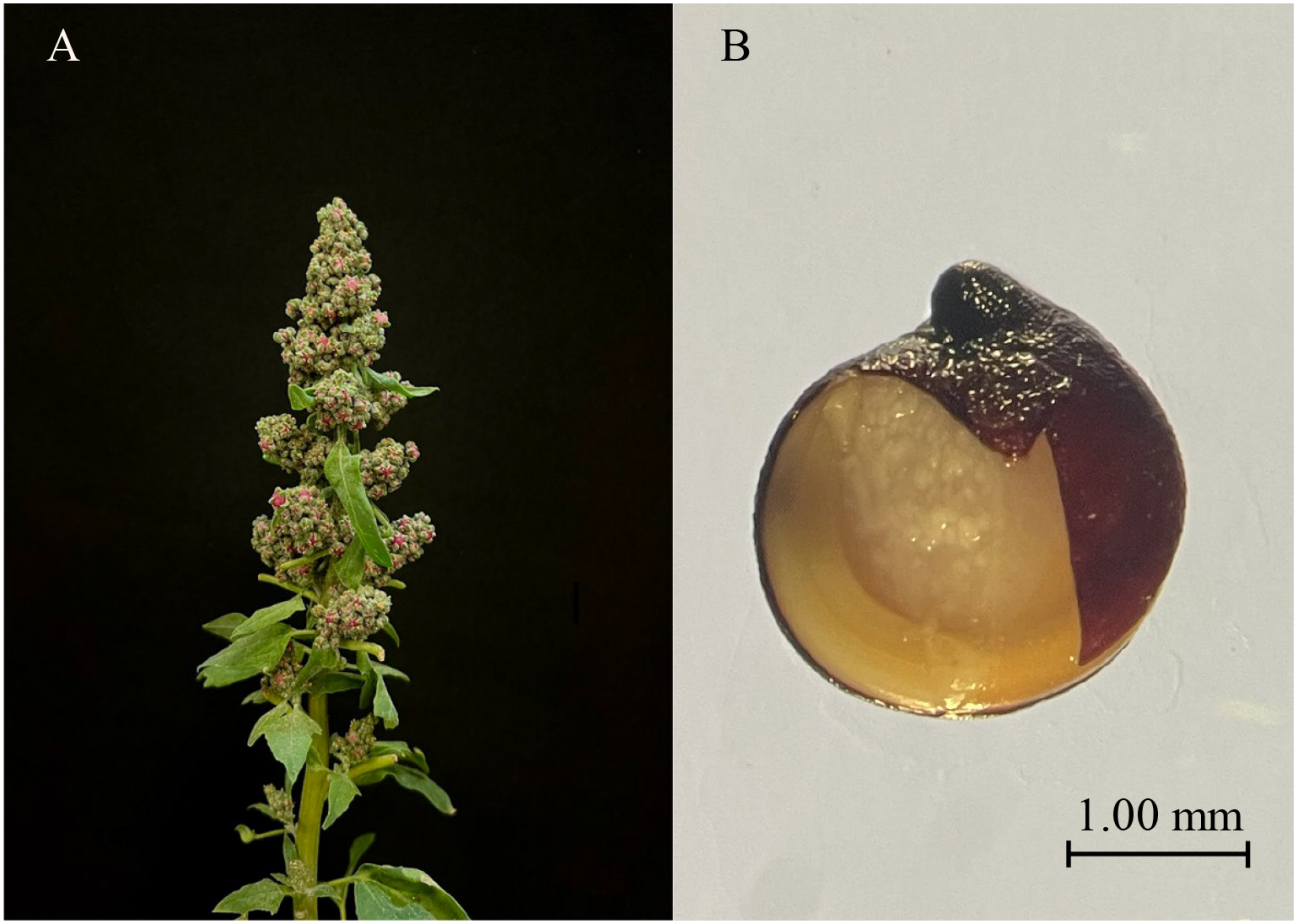
Experimental material CL-2. **(A)** shows the panicle of the CL-2 plant, while **(B)** shows the internal morphology of the CL-2 seed. After removing part of the seed coat, it was captured by a stereomicroscope.

After a thorough assessment of the germination characteristics and PHS resistance of over 30 quinoa varieties conducted by laboratory members in the preliminary stage, the research concluded that no variety has demonstrated robust PHS resistance yet. Nevertheless, variety CL-2 stands out with its high germination percentage, making it highly susceptible to PHS. Consequently, CL-2 was chosen as the focal experimental variety for this study. Quinoa CL-2 (**Figure 1**), sourced from the Key Laboratory of Coarse Cereal Processing, Ministry of Agriculture and Rural Affairs P.R. China. CL-2 seeds were placed in a culture dish with a filter paper, both with a radius of 4.5 cm, and 6 mL of ultrapure water was added. The aim of this research is to establish a theoretical basis for further exploration into quinoa PHS, achieved through an in-depth study of seed germination. Consequently, the cultivation temperature was set in accordance with the average temperature during the quinoa harvesting season in Chengdu, Sichuan Province, China. The seeds were placed in an artificial climate box with a 14-hour light/10-hour dark cycle at 30 °C/24 °C, respectively, and a constant light intensity of 40% during the light period. The experiment lasted for 20 hours under these conditions. Experimental samples were collected at intervals of 4, 8, 12, 16, and 20 hours, as well as from dry seeds at 0 hour. After sampling, the samples were immediately wrapped in tin foil, rapidly frozen in liquid nitrogen, and stored at -80°C for future use.

### 2.2 Determination of germination percentage and water absorption rate

The germination percentage was measured according to the Chinese national standard GB SN/T 0800.14-1999. 100 quinoa seeds were randomly selected and then placed on the soaked filter paper in a culture dish. The seeds were placed in the artificial climate box, and the germination data were recorded. The emergence of the radicle marked the completion of seed germination (Pinto-Irish et al., 2020). Germinated seeds were removed from the culture dish for each record to avoid interference with data statistics, with three biological replicates. The determination of water absorption rate was detailed in prior research study (Gao et al., 2023).

### 2.3 Measurement of physiological and biochemical parameters

The levels of soluble protein, soluble sugar, and starch content were determined according to the instructions of the assay kits provided by Suzhou Comin Biotechnology Co., Ltd. Furthermore, the level of total amylase activity detected based on the instruction of the assay kit provided by Shanghai yuanye Bio-Technology Co., Ltd. High-performance liquid chromatography (HPLC) was employed to determine the content of fructose, glucose, sucrose and maltose during seed germination. The content of ABA and GA_3_ was quantified utilizing the Electron Spray Ionization-High-Performance Liquid Chromatography-Mass Spectrometry/Mass Spectrometry (ESI-HPLC-MS/MS) internal standard method. Enzyme activities for ZEP, NCED, AAO, ABA8’-H, GA20ox, GA3ox, and GA2ox were evaluated employing enzyme-linked immunosorbent assay (ELISA) kits sourced from Jiangsu Meimian Industrial Co., Ltd. Comprehensive details regarding kit models and specific assay methodologies are available in **Appendix A**. All experiments were performed with three biological replicates.

### 2.4 Transcriptome quality control, sequencing, and analysis process

Based on the experimental results in water absorption and germination percentage of quinoa seeds, the timing for collecting samples for transcriptome analysis was determined. The conditions for the transcriptome materials were identical to those described in the “Preparation of Experimental Materials” section. Specifically, selected quinoa seeds were weighed, with 2 g of seeds placed in each petri dish, totaling 18 petri dishes. The samples at 0 hours were directly snap-frozen using liquid nitrogen. The remaining 15 petri dishes were supplied with an equal amount of water (6 mL) to keep the seeds moist and were then placed in an incubator for cultivation. At 4, 8, 12, 16, and 20 hours, three petri dishes were taken from the incubator at each time point, and the seeds were transferred into 3 centrifuge tubes, which were then snap-frozen in liquid nitrogen for transcriptome sampling. Therefore, the experiment involved three biological replicates. Specifically, the cultured seeds were rapidly frozen in liquid nitrogen, and then RNA was extracted according to the instructions of the Plant Tissue RNA Extraction Kit (Tiangen Biotech (Beijing) Co., Ltd.). The integrity of the RNA was evaluated using the RNA Nano 6000 Assay Kit of the Agilent Bioanalyzer 2100 system (Agilent Technologies, CA, USA). Only RNA samples with a RIN (RNA integrity number) exceeding 8.0 were utilized for subsequent cDNA libraries construction. The mRNA with polyadenylation tails was enriched by connecting oligothymidine magnetic beads. The first strand of cDNA was synthesized using fragmented mRNA as a template and random oligonucleotides as primers in the M-MuLV reverse transcriptase system. Subsequently, the RNA strand was degraded by RNase H, and the second strand of cDNA was synthesized using dNTPs (the four deoxyribonucleotide triphosphates) as raw materials in the presence of DNA polymerase I. The purified double-stranded cDNA underwent end-repair, adenine tailing, and ligation of sequencing adapters. cDNA fragments of approximately 250–300 bp were selected using AMPure XP beads, followed by PCR amplification. The PCR products were purified again using AMPure XP beads, ultimately yielding the library. After the completion of the library construction, Qubit2.0 Fluorometer was used for preliminary quantification, and then Agilent 2100 bioanalyzer was used to detect the insert size of the library to ensure its quality for subsequent sequencing. Sequencing by synthesis was then conducted by the Illumina sequencing platform (Novaseq 6000, Illumina, USA).

Transcriptome sequencing was commissioned to Wekemo Tech Group Co., Ltd. (Shenzhen China). Fastp (Chen et al., 2018) was utilized for data quality control (QC), followed by HISAT2 (Kim et al., 2015) which was employed to align the cleaned data with the quinoa reference genome (https://www.cbrc.kaust.edu.sa/chenopodiumdb). Alignment evaluation was carried out through QualiMap v.2.2.2 dev (Okonechnikov et al., 2016). The StringTie software (Pertea et al., 2015) was used to perform transcriptome assembly for each sample individually, followed by the merging of all transcriptome annotations. All transcriptome annotations were compared with the reference genome annotation file using Gffcompare software (Pertea and Pertea, 2020) to predict new transcripts. Gene count, Fragments Per Kilobase of transcript per Million mapped reads (FPKM) and Transcripts Per Million (TPM) were calculated by FeatureCounts (Liao et al., 2014). The DESeq2 package (Love et al., 2014) in the R programming language (version 4.2.2) was used to identify differentially expressed genes (DEGs) during the germination process of quinoa seeds. This identification was based on a model employing the negative binomial distribution. The criteria for determining DEGs were |log2(FoldChange)| > 1 and an adjusted p-value < 0.05.Conduct enrichment analysis on fragments utilizing the R language ClusterProfiler package (Gao et al., 2024), and annotate DEGs through the Gene Ontology (GO), Kyoto Encyclopedia of Genes and Genomes (KEGG), and STRING databases.

### 2.5 Searching for key genes

The Spearman correlation coefficient of DEGs residing within distinct biological pathways was calculated by the R language. Gene correlations were assessed using a threshold coefficient of ≥ 0.4 and a significance level of p < 0.05. Furthermore, the correlation network was visualized by the cytoNCA plugin within the Cytoscape.

### 2.6 RNA extract and RT-qPCR

The sample RNA was successfully extracted by following the detailed instructions outlined in the plant tissue extraction kit (Tiangen, Beijing, China). Subsequently, after confirming the RNA’s quality through rigorous inspection, cDNA synthesis was carried out in accordance with the instructions provided in the FastKing RT Kit (with gDNase, Tiangen, Beijing, China). Detailed procedures pertaining to RNA quality assessment, cDNA synthesis, and primer design can be found in previous research (Gao et al., 2023).

The reference gene for RT-qPCR standardization was *actin* (ACT) (Gao et al., 2023). RT-qPCR was performed by M5 Hiper SYBR Premium EsTaq with Tli RNaseH (MeiBio, Beijing, China). The RT-qPCR reaction mixture included 1 µL of cDNA (80 ng/µL), 0.4 µL of forward primer (100 µM), 0.4 µL of reverse primer (100 µM), 10 µL of 2 × M5 Hiper SYBR Premium EsTaq (containing Tli RNaseH), and RNase Free ddH_2_O (adjusted to 20 µL). The RT-qPCR reaction consisted of an initial pre-denaturation step at 95 °C for 30 s, followed by denaturation at 95 °C for 5 s, and extension at 60 °C for 30 s, repeated for 40 cycles. The specificity of primer amplification was validated through a melting curve analysis ranging from 60 to 95 °C with a ramp rate of 5 °C/s.

### 2.7 Statistical analysis

The experimental data were curated using Excel 2016 (Microsoft, USA). Data were analyzed using SPSS 26 (IBM Co. Armonk, NY, USA). Based on the normal distribution of the data, one-way ANOVA was used to determine the variations in different indicators across various time periods. The Warren-Duncan ‘s test was used for multiple comparisons (P < 0.05). Graphical representations were created by GraphPad Prism 8 (GraphPad Software, USA), Origin Pro 2023 (OriginLab Northampton, Massachusetts, USA.), Cytoscape (Cytoscape software, USA), and Adobe Illustrator 2023 (Adobe Co., USA).

## 3 Results

### 3.1 Germination characteristics and water uptake

**Figure 2:**
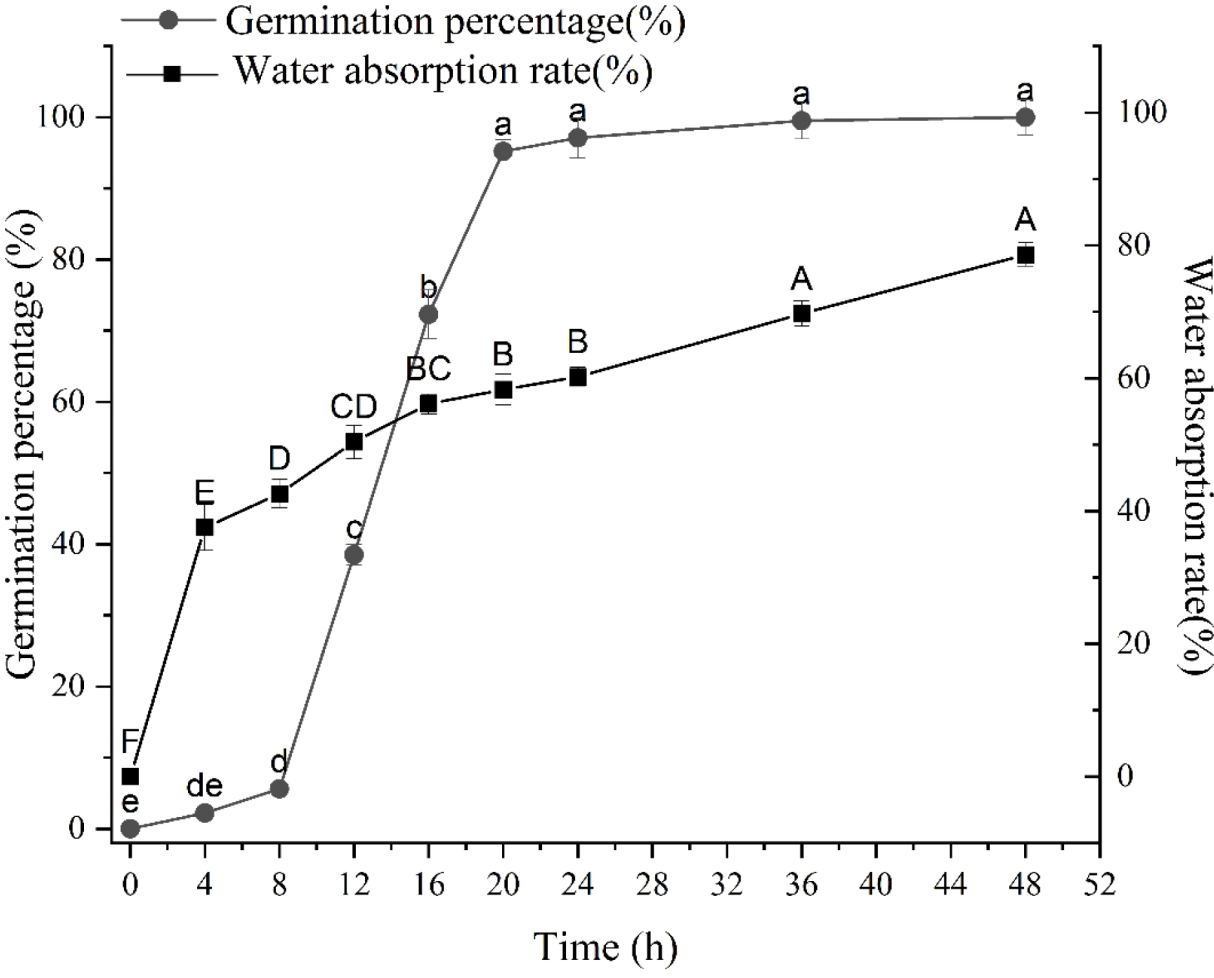
Changes in germination percentage and water absorption rate of seeds during germination process. Uppercase and lowercase letters indicate significant differences among different period in the same treatments using Waller-Duncans test (P < 0.05). Uppercase letters represent the significance of water absorption rate, while lowercase letters represent the significance of germination percentage.

The germination percentage of CL-2 seeds over a 48-hour period (**Figure 2**) displayed a gradual increase from 0 to 20 hours, with no statistically significant changes observed thereafter until 48 hours (p > 0.05). As a result, the optimal time for sampling biochemical indicators in the later stage was determined to be at the 20-hour mark. The germination percentage exhibited relatively low levels during the initial 0–8 h, referred to as the “germination preparation stage”, followed by the 8–20 h characterized as the “germination stage”, and the subsequent 8–16 h designated as the “rapid germination stage”. After 20 hours, the average germination percentage of seeds reached 95%, suggesting optimal seed viability. The findings regarding seed water absorption rate (**Figure 2**) demonstrated a swift uptake of water by the seeds within the initial 4 hours, labeled as the “rapid water absorption stage”, followed by a gradual and consistent water absorption starting at 8 hours.

### 3.2 Changes of storage compounds during seed germination

**Figure 3:**
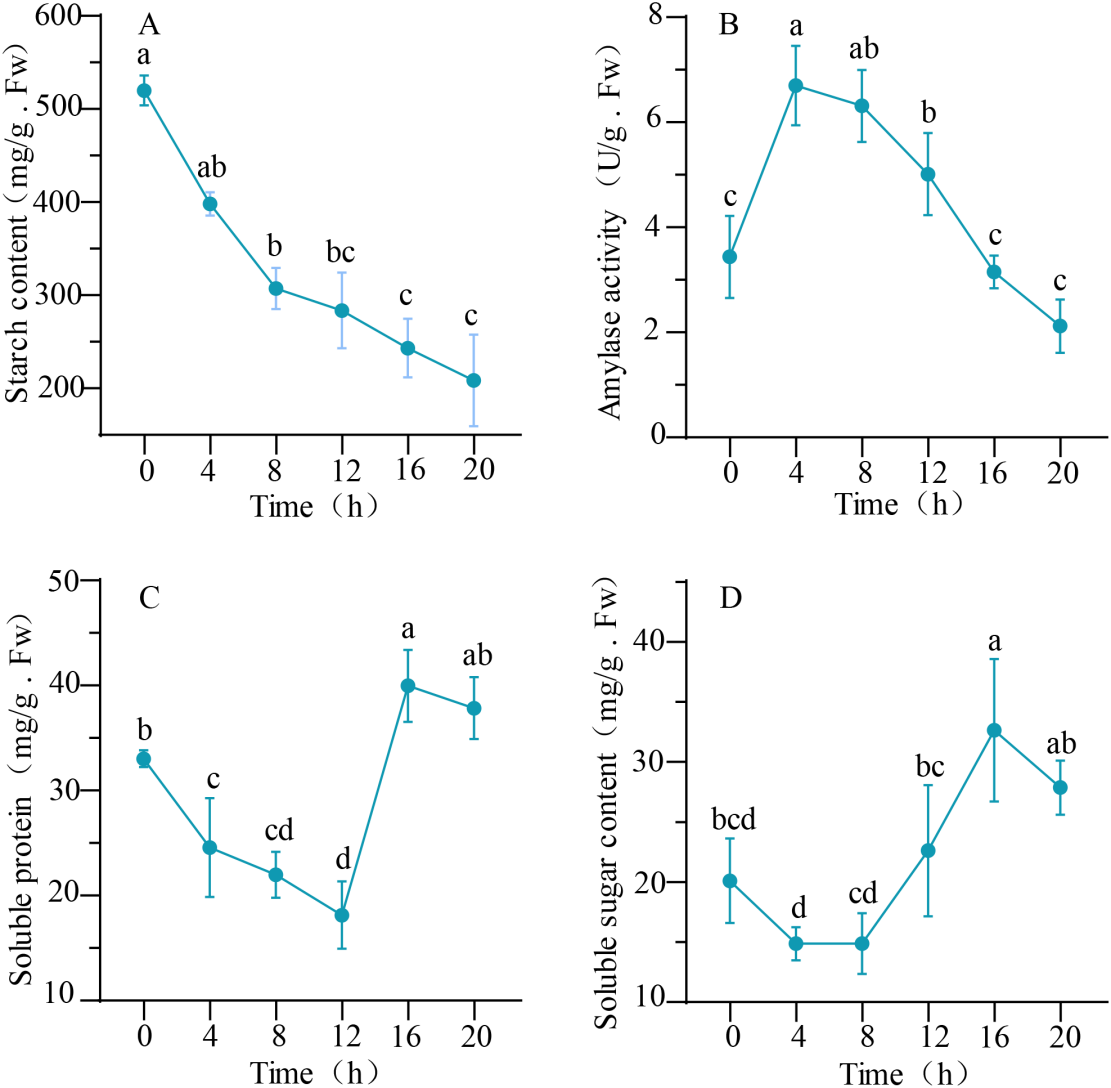
The dynamic changes in physiological and biochemical indicators during the germination process of quinoa seeds. **(A, C, D)** represent the content of starch, soluble protein, and soluble sugar. While **(B)** represent the amylase activity. Different letters indicate significant differences among different period in the same treatments using Waller-Duncans test (P < 0.05).

A reduction in starch content was observed during the germination process of seeds, as illustrated in **Figure 3**. The highest starch content, recorded at 519.85 ± 13.14 mg/g, was observed in 0-hour. Following 20 hours of germination, the starch content decreased to its lowest point, measuring at 208.66 ± 40.19 mg/g. Changes in total amylase activity were also depicted in **Figure 3**. During the rapid water absorption stage (0–4 h), amylase activity peaked at 6.70 ± 0.62 U/L. Subsequently, there was a decline in amylase activity, with the enzyme activity reaching its minimum at 2.12 ± 0.42 U/L after 20 hours. The soluble protein content demonstrated a decrease from 0 hour to 12 hours, followed by a subsequent overall increase in the later stages. Specifically, at the 12-hour mark, the minimum value observed was 8.13 ± 2.62 mg/g, which subsequently peaked at 39.96 ± 2.79 mg/g by the 16-hour mark. In contrast, the soluble sugar content exhibited a decline during the initial 0–8 h period, followed by a rapid increase from 8 hours to16 hours, and a subsequent decrease after 16 hours. The minimum and maximum soluble sugar content values were recorded at 14.86 ± 1.12 mg/g and 32.64 ± 4.85 mg/g, respectively, at 4 and 16 hours.

### 3.3 Changes of small molecule sugar content during seed germination

**Figure 4:**
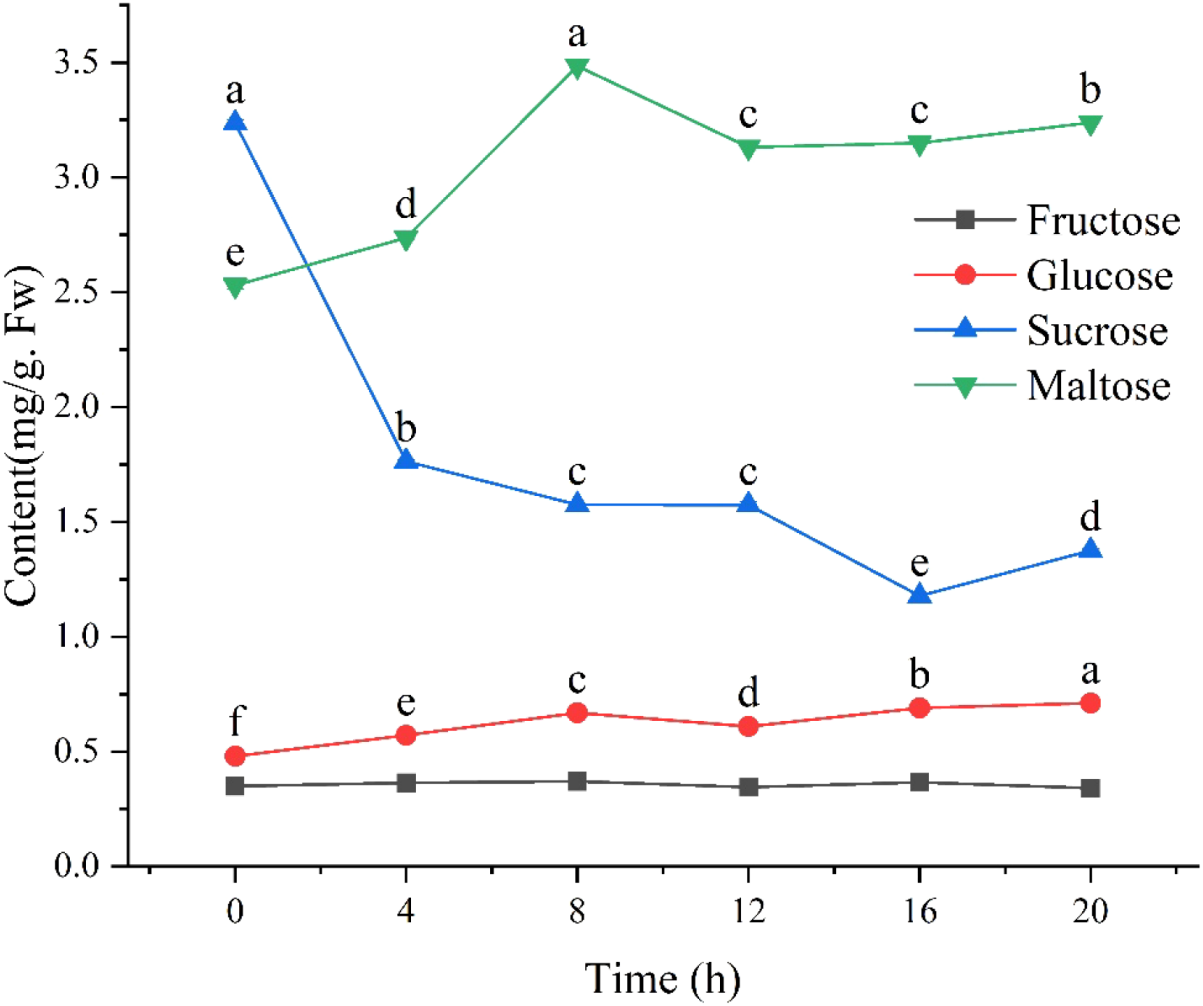
Dynamic fluctuations in the content of low-molecular-weight sugars during the germination of quinoa seeds. Different letters indicate significant differences among different period in the same treatments using Waller-Duncans test (P < 0.05).

The results of analyzing fructose, glucose, sucrose, and maltose levels during the germination process of quinoa seeds are presented in **Figure 4**. In terms of the sugars analyzed, fructose displayed the lowest content, fluctuating within a narrow range of 0.335 to 0.358 mg/g. In contrast, glucose levels showed an upward trend during the initial stages of germination (0–8 h), indicating a consistent increase in concentration over time. When compared to monosaccharides, disaccharides demonstrated higher levels, specifically maltose with a distribution spanning from 2.52 to 3.474 mg/g and sucrose distribution ranging from 1.177 to 3.22 mg/g. The maximum value of maltose content was observed at 8 hours. Sucrose, on the other hand, was higher in dry seeds but underwent a marked decline throughout the germination process.

### 3.4 Changes in ABA and GA3 during germination process

**Figure 5:**
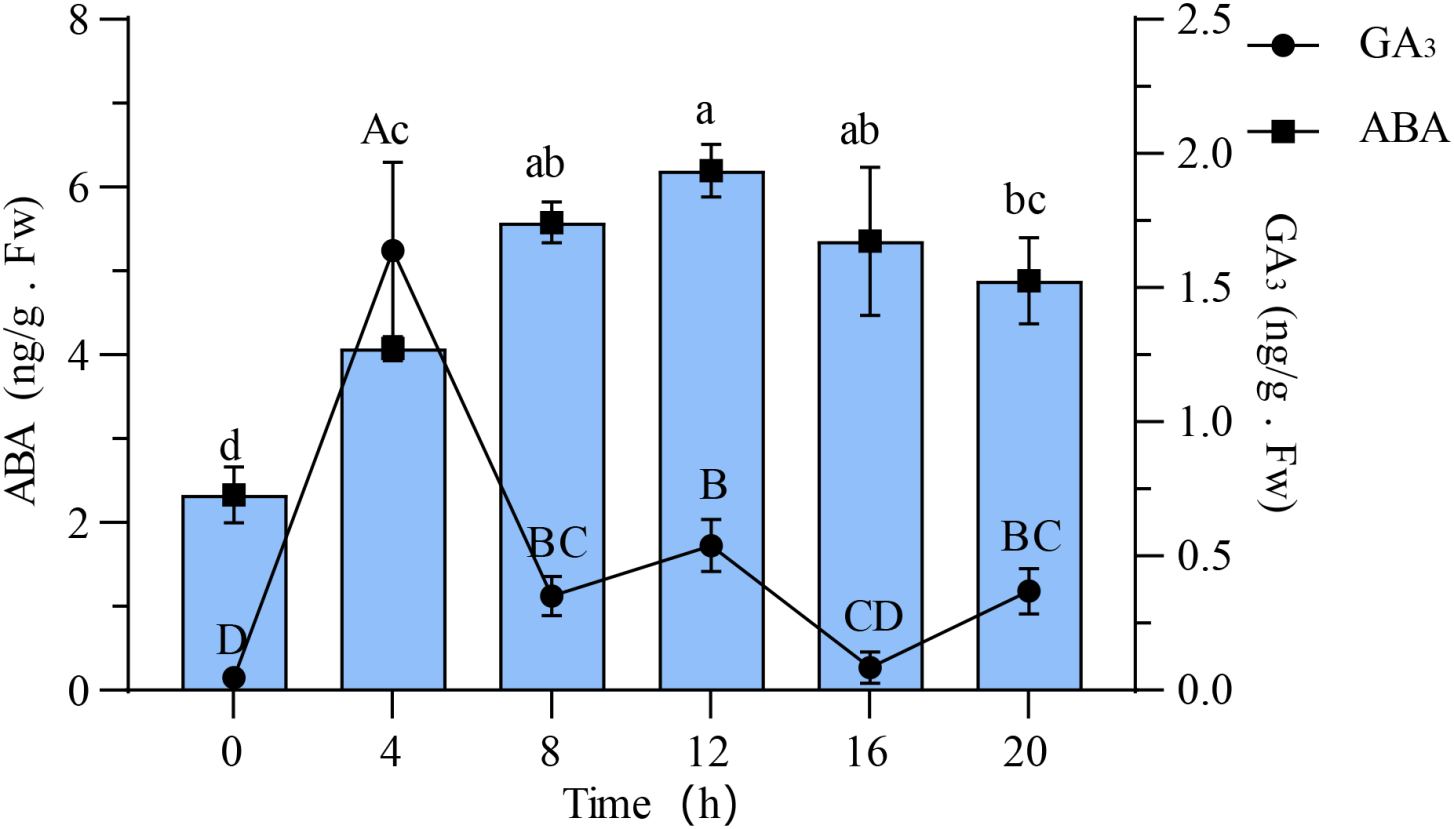
Variations in ABA and GA_3_ concentrations throughout the germination process of quinoa seeds. Different letters indicate significant differences among different period in the same treatments using Waller-Duncans test (P < 0.05). Lowercase letters indicate significant differences in ABA, whereas uppercase letters denote significant differences in GA_3_ concentrations using the same statistical method.

The results of ABA and GA_3_ content within 20 hours of seed germination are depicted in **Figure 5**. A notable pattern was observed in the ABA content, showing an increase followed by a decline at the 12-hour mark. The lowest ABA content was detected in the dry seeds, measuring at 2.33 ± 0.27 ng/g, whereas the peak level was recorded at 12 hours, reaching 6.20 ± 0.26 ng/g. Statistical analysis indicated a significant difference in ABA content at 0 hour, 4 hours, and 8 hours (p < 0.05), with no significant disparity observed between the subsequent time intervals from 8 to 20 hours (p > 0.05). On the contrary, the GA_3_ content in the dry seeds was observed to be at its lowest level (0.05 ± 0.02 ng/g), during the stage of rapid water absorption by the seeds, at the 4-hour mark, the GA_3_ concentration reached its peak at 1.64 ± 0.27 ng/g before experiencing a sharp decline. Between the 8 to 20-hour time interval, the GA_3_ content fluctuated within the range of 0.04 to 0.65 ng/g.

### 3.5 Changes in ABA and GAs-related enzyme activity

**Figure 6:**
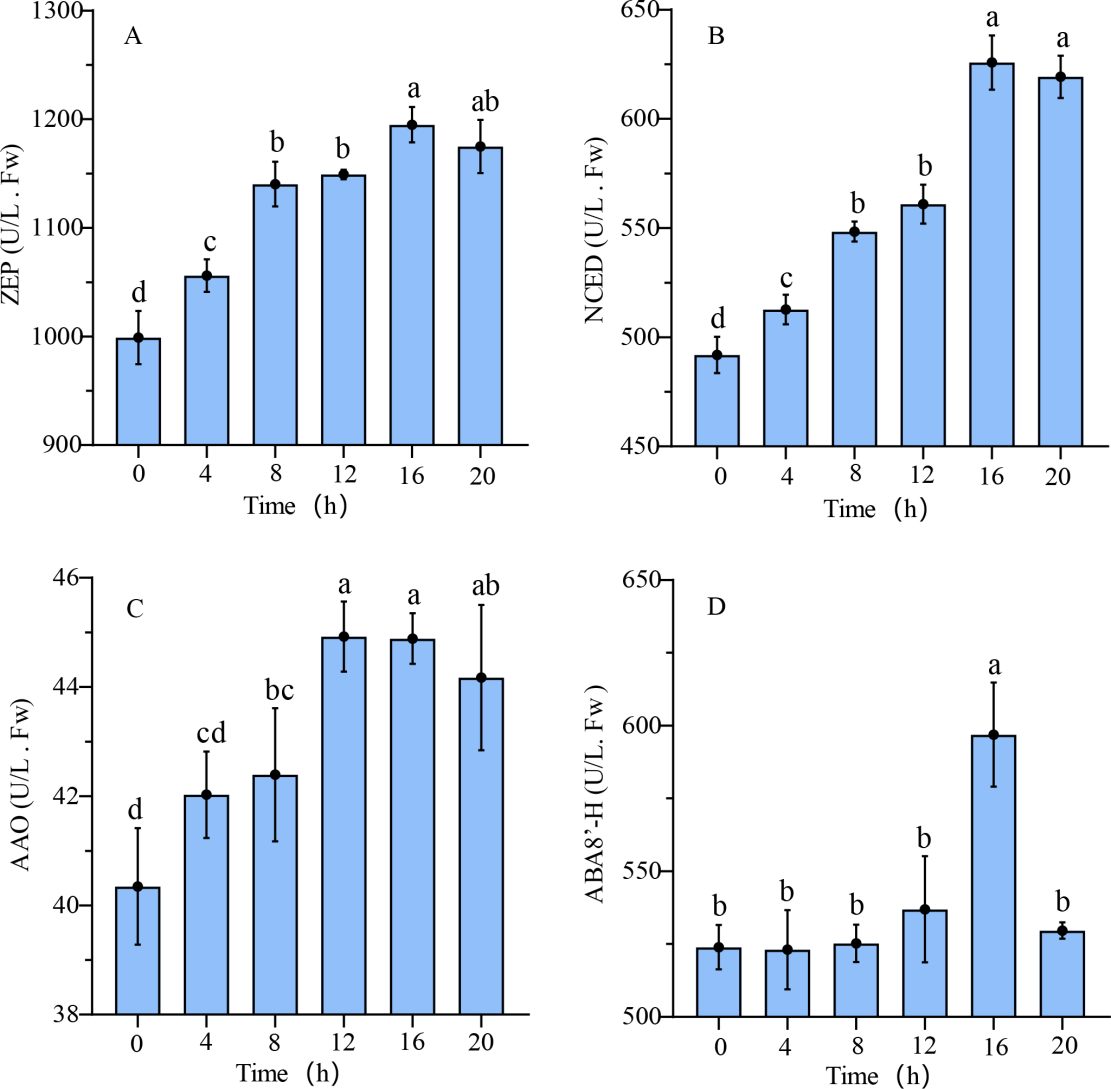
ABA synthesis and metabolic enzyme activity during the germination process of quinoa seeds. **(A-D)** represent the enzyme activities of ZEP, NCED, AAO, and ABA8’-H during seed germination, respectively. Different letters indicate significant differences among different period in the same treatments using Waller-Duncans test (P < 0.05).

The results of hormone-related enzyme activity are illustrated in **Figure 6**. The enzyme activity linked to ABA synthesis exhibited the lowest value in the dry seeds, with ZEP, NCED, and AAO showing minimum enzyme activities of 998.91 ± 20.01 U/L, 491.92 ± 6.81 U/L, and 40.43 ± 0.87 U/L, respectively. Subsequently, during the rapid germination stage of seeds, the enzyme activity peaked at 16 hours, 16 hours, and 12 hours, respectively, with values of 1194.87 ± 13.33 U/L, 625.77 ± 10.11 U/L, and 44.93 ± 0.52 U/L. The ABA synthase ZEP demonstrated the highest level of activity, ranging from 979.39 to 1210.35 U/L, while the AAO enzyme exhibited the lowest activity, with a range of 39.49 to 45.68 U/L. The ABA8’-H enzyme, which mediates ABA decomposition, exhibited consistent activity during the germination preparation phase, followed by a rapid increase and subsequent decline around the 16-hour mark.

**Figure 7:**
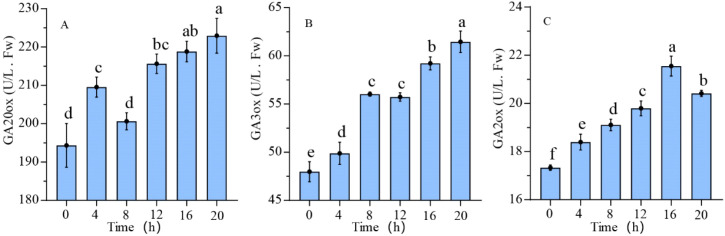
GAs synthesis and metabolic enzyme activity during the germination process of quinoa seeds. **(A-C)** represent the enzyme activities of GA20ox, GA3ox, and GA2ox during seed germination, respectively. Different letters indicate significant differences among different period in the same treatments using Waller-Duncans test (P < 0.05).

The expression levels of GA20ox and GA3ox, key enzymes involved in GAs synthesis, exhibited a consistent upward trajectory during seed germination, as depicted in **Figure 7**. In the dry seeds, the enzymatic activities of GA20ox and GA3ox were relatively low at 194.33 ± 4.66 U/L and 47.97 ± 0.85 U/L, respectively. Subsequently, from 8 hours to 20 hours post-germination, the enzymatic activities of both synthases increased steadily, reaching maximum values of 222.94 ± 3.70 U/L and 61.46 ± 0.91 U/L for GA20ox and GA3ox, respectively. The activity of GAs metabolic enzyme GA2ox displayed a biphasic pattern, with an initial increase followed by a decline, reaching a maximum value of 21.55 ± 0.34 U/L at 16 hours. The lowest activity of GA2ox was observed in the dry seeds, registering at 17.33 ± 0.09 U/L.

### 3.6 Illumina sequencing and quality control

Based on the experimental results of germination rates, we observed that 8 hours marked a turning point in germination. Prior to 8 hours, the germination rate was relatively low. However, once exceeding this point, the seed germination rate accelerated significantly. Based on this finding, time points before and after 8 hours (specifically, 4 hours and 12 hours) were decided to be selected as comparisons for pre- and post-germination stages. Quinoa seeds underwent a germination period of 4 hours for the control group and 12 hours for the experimental group. The specifics of the sequencing samples after QC are presented in **Appendix Table 1**. Following QC, the data volume of clean data per sample ranged from 6.04 GB to 6.47 GB, with an overall comparison rate of 96.70% to 97.50%. The Q30 ratio ranged from 92.44% to 93.12%, and the GC content varied from 43.69% to 44.85%. These results confirm the reliability of the transcript data and the use of clean data for further analyses.

Furthermore, by assessing the correlation between samples using TPM data, the reproducibility of biological experiments within the sample group was evaluated. The Spearman correlation coefficient analysis revealed that coefficients greater than 0.92 indicate a strong correlation between samples, as depicted in **Appendix Figure 1**.

### 3.7 DEGs analysis by GO annotation

**Figure 8:**
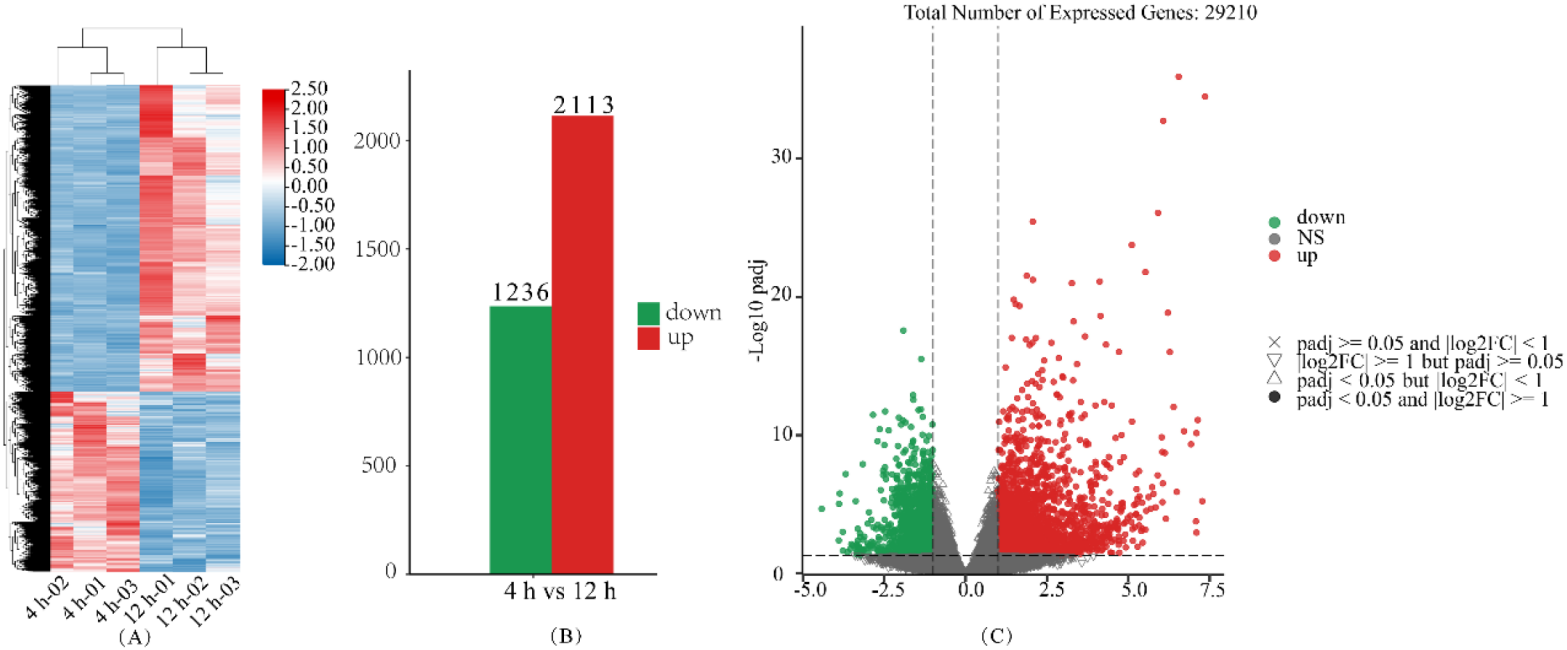
Transcriptome differential gene expression. **(A-C)** represent differential gene analysis expression heatmaps, differential gene analysis summary bar charts, and differential gene analysis volcano charts, respectively.

By utilizing the criteria of log2(FoldChange) > 1 and padj < 0.05 to identify DEGs, a total of 3349 DEGs were identified in the comparison between 4 hours and 12 hours; of these, 2113 DEGs exhibiting upregulation and 1236 DEGs showing downregulation (**Figure 8**). The functional annotation of DEGs within specific pathways was performed using GO, resulting in a total of 3030 GO entries. These entries encompassed 2132 biological processes, 595 molecular functions, and 303 cellular components. A total of 15686 genes were associated with these GO entries, specifically including 1588 DEGs included. The *p*-values associated with these annotations were adjusted using the false discovery rate (FDR) method.

**Figure 9:**
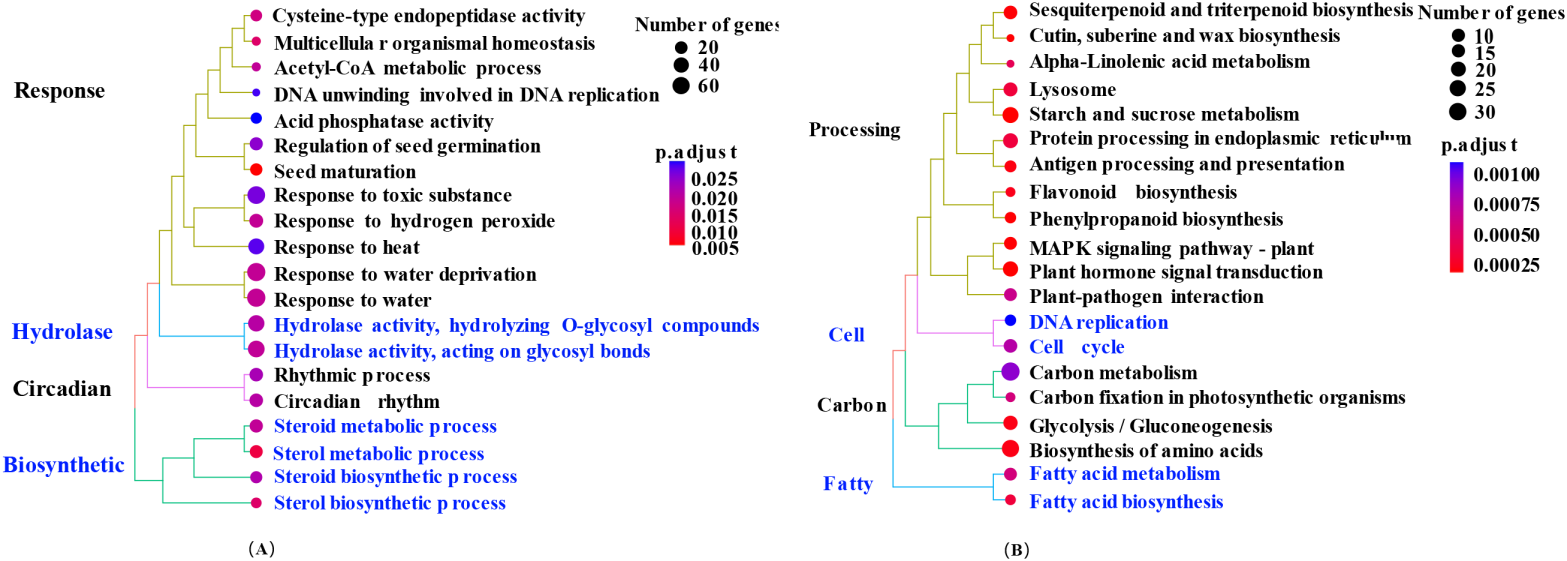
The top 20 significantly enriched pathways of differentially expressed genes. **(A, B)** represent significantly enriched pathways for GO and KEGG, respectively.

In the top 20 most significant entries of GO analysis (**Figure 9A**), the focus is on Response, Hydrolase, Circadian, and Biosynthetic. The top 5 significant biological processes include Seed maturation (0010431), Sterol metabolic process (00016125), Multicellular organismal homeostasis (00048871), Sterol biosynthetic process (0016126), and Response to water (00009415). In terms of molecular functions, the most significant entries are Cysteine-type endopeptidase activity (0004197), Hydrolase activity and acting on glycosyl bonds (0016798), Hydrolase activity and hydrolyzing O-glycosyl compounds (0004553), and Acid phosphatase activity (0003993).

Among statistically significant GO terms (p < 0.05), which pertaining to seed germination and maturation, entries included Seed maturation (0010431), Regulation of seed germination (0010029), Rhythmic process (0048511), Positive regulation of seed germination (0010030), and Regulation of seedling development (1900140). In the carbohydrate-related GO entries, Carbohydrate catabolic process (0016052) exhibited statistical significance with an adjusted p-value of 0.0447.

### 3.8 DEGs analysis by KEGG annotation

To further investigate the DEGs expression profiles, the DEGs were annotated by the KEGG database. A total of 13242 genes were enriched in 365 KEGG pathways, including 1639 DEGs. The findings presented in **Figure 9B** illustrate the top 20 pathways with the lowest adjusted p-values, suggesting a notable enrichment in pathways associated with carbon metabolism, plant hormone signaling, biosynthesis of biologically active compounds, and lipid-related processes. To further elucidate the alterations in starch, sugar, ABA, and GA_3_ levels, as well as signaling pathways related to ABA and GAs during quinoa seed germination, an extensive annotation analysis of the KEGG pathways was conducted.

**Figure 10:**
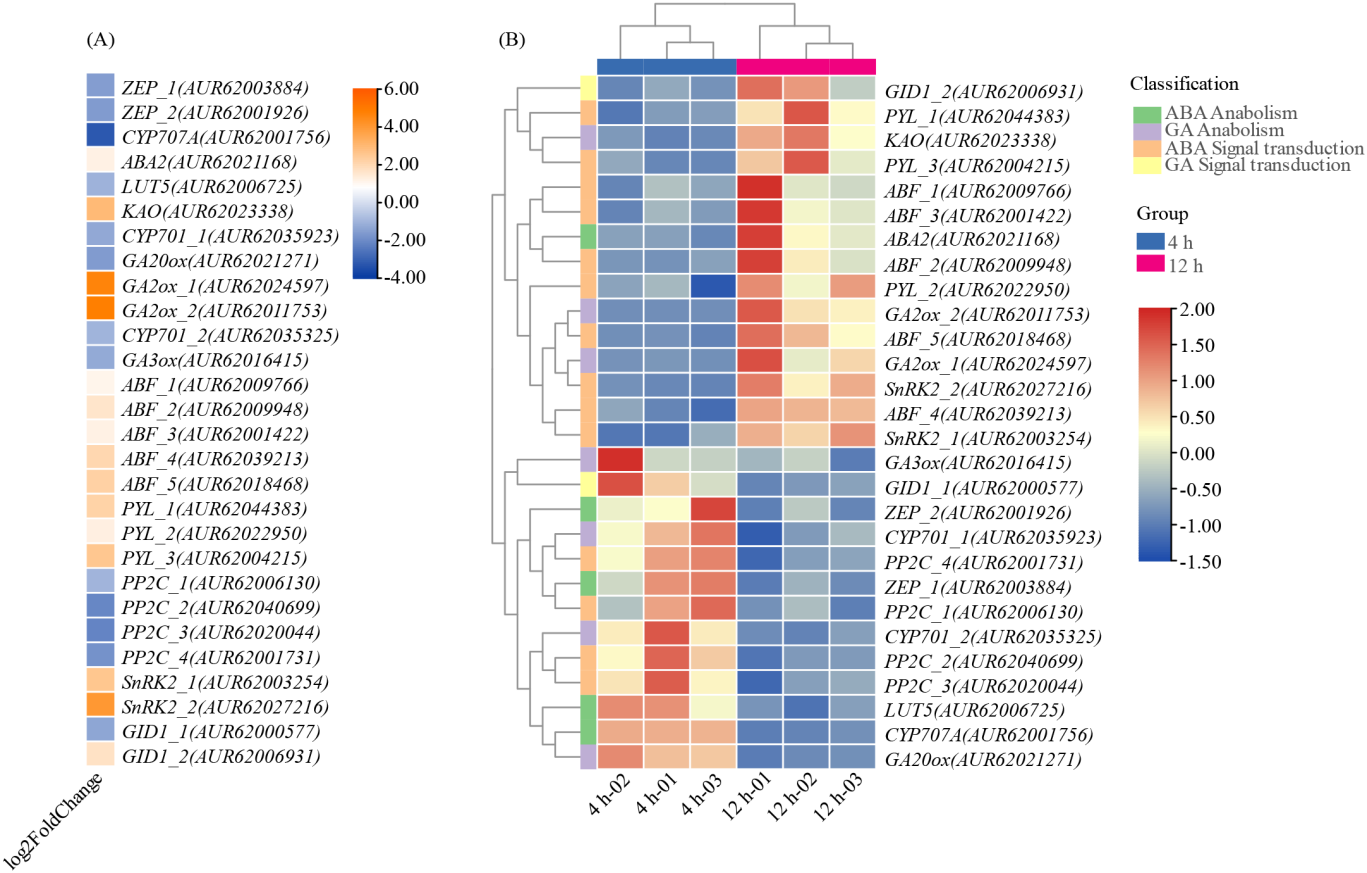
Changes in FPKM and FC of ABA and GAs differentially expressed genes. **(A)** FC; **(B)** FPKM.

The result revealed that the ABA synthesis metabolic pathway (M00372) did not show statistically significant enrichment (p > 0.05), while the Diterpenoid biosynthesis pathway (ko00904), including the GAs biosynthetic pathways M00927, M00928, and M00929, displayed significant enrichment (p < 0.05). Furthermore, the Plant hormone signal transduction pathway (ko04075) exhibited highly significant enrichment (p < 0.001). The expression levels of DEGs, as measured by Fragments Per Kilobase Million (FPKM) and Fold Change (FC), related to the ABA and GAs pathways are illustrated in **Figure 10**.

In the realm of hormone synthesis metabolism, the ABA pathway consists of a total of 21 genes, among which 5 genes, namely *ZEP_1*, *ZEP_2*, *CYP707A*, *ABA2*, and *LUT5*, were identified as DEGs (The full names and IDs corresponding to the abbreviations of genes are detailed in **Appendix B**). The FC for ABA biosynthesis genes varied from -1.68 to 1.22 overall, with *ZEP* and *LUT5* showing downregulation in expression, while *ABA2* exhibited upregulation. Notably, the metabolic gene *CYP707A* displayed significant downregulation, with a FC of -3.25. Meanwhile, the pathways related to ABA and GAs were exhibited in **Appendix Figure 2**.

Furthermore, a total of 21 genes have been identified as being associated with GAs, with 7 of these genes identified as DEGs: *CYP701_1*, *CYP701_2*, *GA3ox*, *KAO*, *GA20ox*, *GA2ox_1*, and *GA2ox_2*. Specifically, genes *GA2ox_1*, *GA2ox_2*, and *KAO* exhibited significant upregulation during germination, while *CYP701_1*, *CYP701_2*, *GA3ox*, and *GA20ox* showed significant downregulation. The observed upregulation of the upstream GAs synthesis gene *KAO* (FC = 2.96) and the downregulation of *CYP701_1*, *CYP701_2*, *GA20ox*, and *GA3ox* (with FC ranging from -1.60 to -1.01) suggest a regulatory role in GAs synthesis. Noteworthy is the differential expression of DEGs of *GA2ox* in quinoa, specifically *GA2ox_1* and *GA2ox_2*, with FC of 4.69 and 4.85, respectively.

Additionally, 48 genes are involved in the ABA signaling pathway and 13 genes in the GAs signaling pathway within the broader context of plant hormone signaling pathways. Among these, 14 DEGs were identified as ABA signaling genes, including *SnRK2_1*, *SnRK2_2*, *PP2C_1*, *PP2C_2*, *PP2C_3*, *PP2C_4*, *ABF_1*, *ABF_2*, *ABF_3*, *ABF_4*, *ABF_5*, *PYL_1*, *PYL_2*, and *PYL_3*. Significantly, a total of 10 genes, including *ABF*, *PYL*, and *SnRK2*, showed upregulation, while *PP2C* displayed downregulation. Moreover, two DEGs, namely *GID1_1* and *GID1_2*, were identified within the GAs signaling pathways, with *GID1_2* being upregulated and *GID1_1* being downregulated.

**Figure 11:**
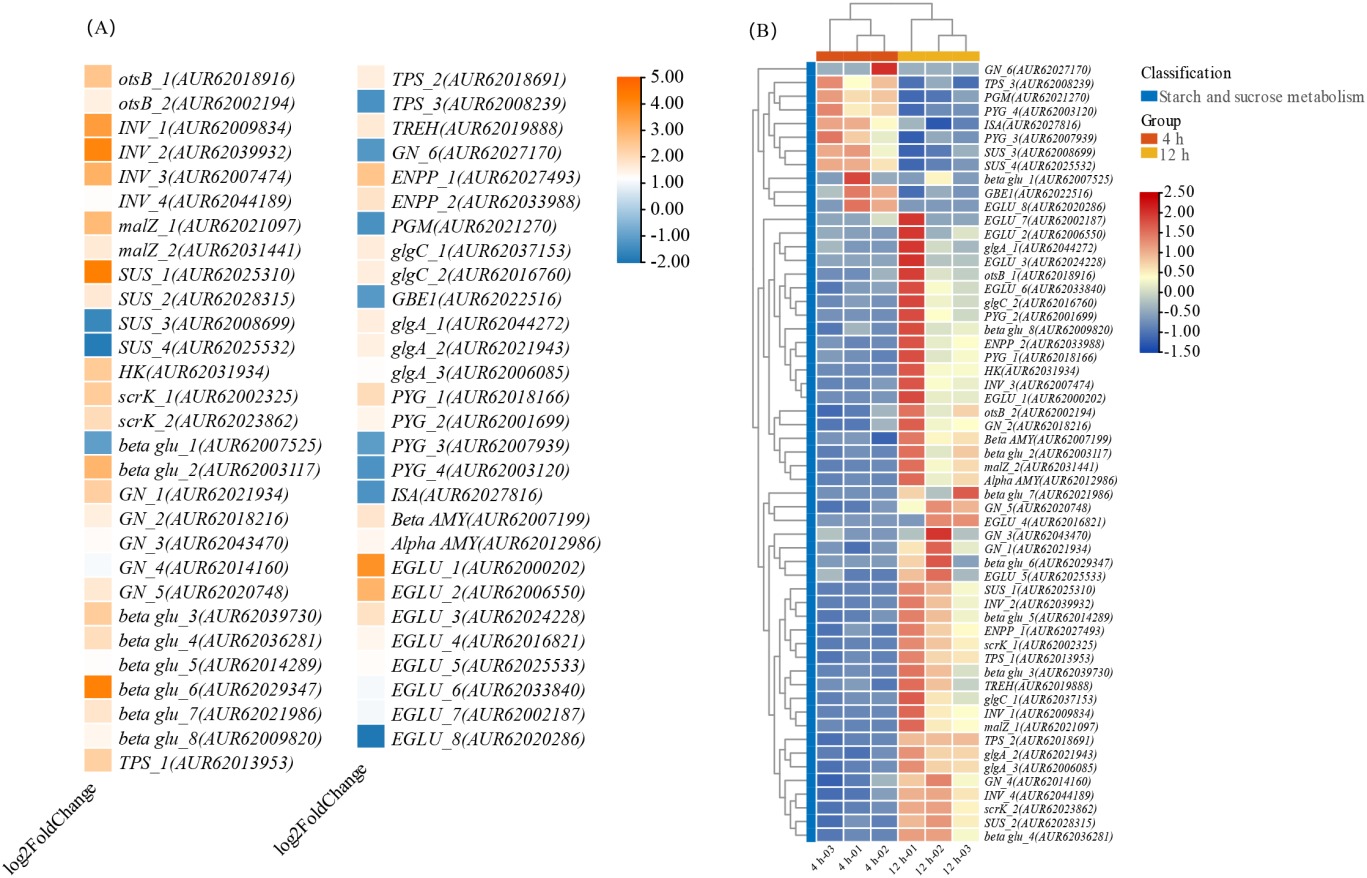
Changes in FC and FPKM genes for starch and sucrose metabolism differences. **(A)** FC; **(B)** FPKM.

The starch and sucrose metabolism pathway (ko0050) exhibited significant enrichment, encompassing a total of 236 genes, of which 57 were identified as DEGs (**Figure 11**). The schematic representation of the starch and sucrose metabolism process is depicted in **Appendix Figure 3**, demonstrating the breakdown of sucrose into D-fructose and D-glucose by *INV* and *malZ*, as well as the interconversion of sucrose with UDP-glucose by *SUS* genes. Four homologous *SUS* genes were identified in quinoa, with *SUS_1* and *SUS_2* showing upregulation by 4.27 and 1.69 times, respectively, while *SUS_3* and *SUS_4* downregulated, with FC of -1.56 and -1.84, respectively. In quinoa, four homologous genes of *INV* were identified among DEGs, with the highest FC of 4.12. The expression levels of the genes involved in D-fructose degradation metabolism, such as *HK* and *scrk* were concurrently upregulated.

In addition to the breakdown of sucrose into glucose and maltose by *malZ*, and cellulose into glucose by *EGLU* and *β-glu*, a total of 8 *EGLU* homologous genes and 8 *β-glu* homologous genes were identified in quinoa. Among these, only *EGLU_8* showed downregulation with an FC of -1.93. Quinoa also possesses 6 homologous genes of *β-glu*, all of which were upregulated.

Simultaneously, starch undergoes enzymatic catalysis by AMY to produce maltose, while AMY is also involved in the hydrolysis of starch into dextrin. The levels of expression for both *beta AMY* and *alpha AMY* were elevated. In the process of starch biosynthesis, D-glucose-1P is converted into ADP glucose by *glgC*, subsequently leading to the formation of amylose through the activity of the starch synthase gene *glgA*, resulting in starch formation under the influence of *GBE1*. The expression levels of *glgC* and *glgA* increased during germination, whereas the expression of *GBE1* decreased with an FC of -1.16.

**Figure 12:**
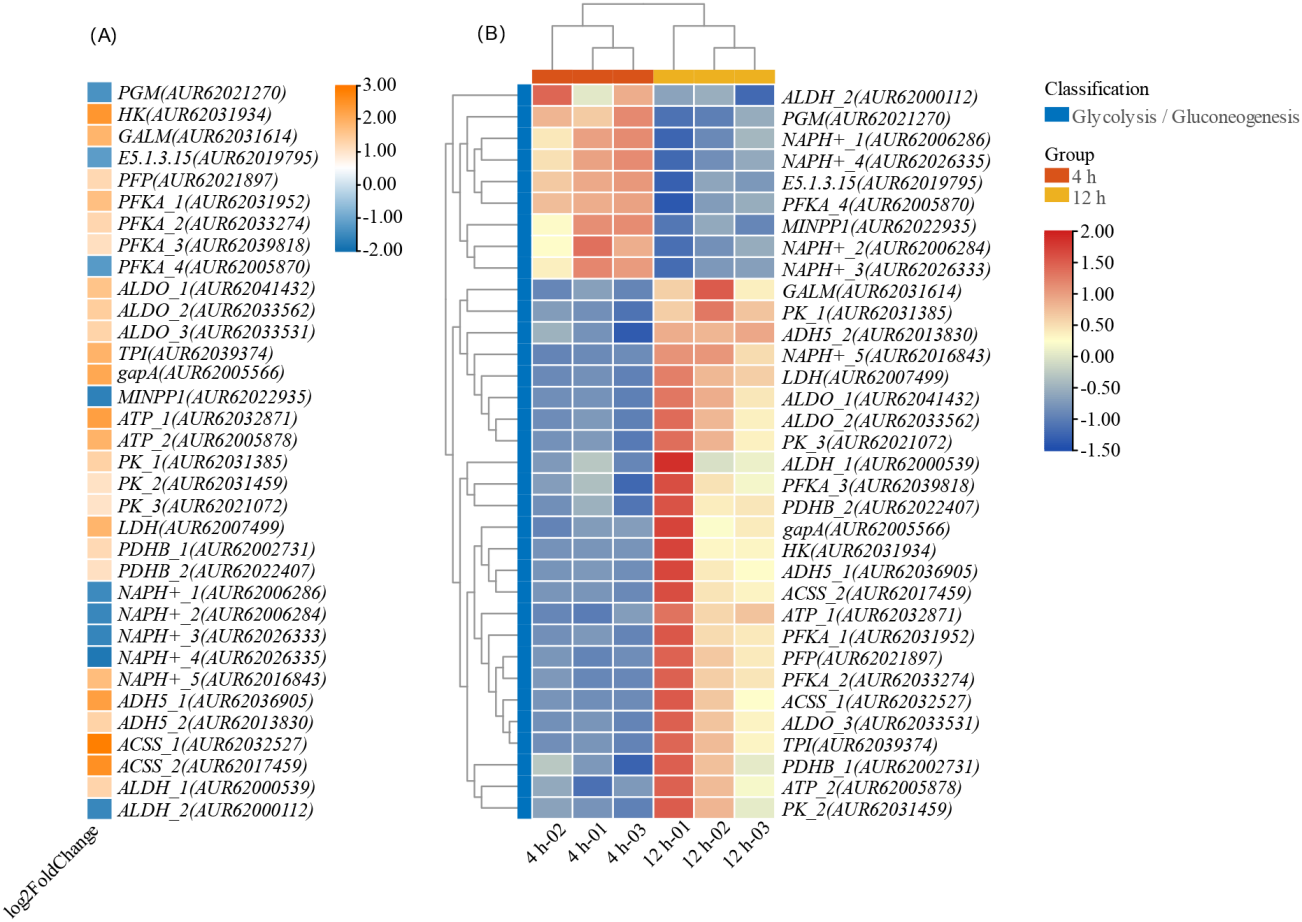
Changes in differential genes FC and FPKM in glycolysis. **(A)** FC; **(B)** FPKM.

The analysis revealed that 161 genes were involved in the glycolysis/gluconeogenesis pathway (ko00010), with 34 DEGs identified (**Figure 12**). The genes involved in the glycolysis/gluconeogenesis pathway are presented in **Appendix Figure 4**. In the initial phase of glycolysis, commonly known as the “energy consumption phase,” glucose phosphorylation facilitated by *GALM* and *HK* results in the production of α-D-glucose-6-phosphate. Subsequently, α-D-glucose-6P was isomerized to β-D-fructose-6P and further phosphorylated to generate β-D-fructose 1,6-bisphosphate through the actions of *PFKA* and *PFP*. *PFKA* encodes a crucial enzyme that regulates the rate of glycolysis, and in quinoa, 4 homologous genes (*AUR62031952*, *AUR62033274*, *AUR62039818*, *AUR62005870*) were identified. Among these, only *AUR62005870* exhibited downregulation, with a FC of -1.2.

The enzyme ALDO facilitated the condensation of a six-carbon molecule into two three-carbon compounds, glycerone-P and D-glyceraldehyde 3-P. These three-carbon compounds can be interconverted by the gene *TPI*. In the second stage of glycolysis, referred to as the “energy -yielding stage,” the conversion of three-carbon compounds into 3-phospho-D-glyceroyl phosphate by the gene *gapA*. Subsequently, the enzymes MINPP1, PK_1, and PK_2 facilitated the production of pyruvate, ultimately completing the glycolytic pathway.

Pyruvate was further metabolized through anaerobic oxidization to lactate by LDH, or alternatively underwent decarboxylation to generate Acetyl CoA and ethanol, among others byproducts. Acetyl CoA then entered the Tricarboxylic acid cycle (TCA cycle) for further energy production. The process of pyruvate salt formation was facilitated by enzymes including *PDHB*, *NAPH^+^*, *FRMA*, *ACSS1_2*, and *ALDH*. Notably, the expression levels of *ACSS1_2*, *FRMA*, and *PDHB* were increased, whereas *NADH^+^* levels were decreased.

### 3.9 Results of key genes

Utilizing the R language and Cytoscape, betweenness centrality was computed to identify pivotal genes within the target pathway. Out of the 28 nodes encompassed in the ABA and GAs-related pathways, the top 3 genes were identified as *SnRK2_1*, *GA2ox_2*, *ABF_2* (**Appendix Figure 5**). Additionally, the associations between starch and sucrose metabolism pathways and glycolysis pathways individually assessed, leading to the establishment of networks for the top 30 correlated nodes within these pathways (**Appendix Figures 6, 7**). The findings revealed that *malZ_2*, *glgA_2*, and *SUS_3* were the top 3 ranked genes in the starch and sucrose metabolism pathway, while *PDHB_2*, *ALDH_1*, and *HK* were the top 3 in the glycolysis pathway.

Based on the rankings provided by 9 different computation methods in Cytohubba, the intersection of genes ranked by these diverse methods was chosen, and these genes were designated as the central genes calculated by Cytohubba’s computation. The results showed that *GA3ox*, *GA20ox*, *ABA2*, *CYP707A*, and *ZEP* as central genes in the ABA and GAs-related gene pathways. However, in the realm of starch and sucrose metabolism, a total of 5 central genes were identified: *SUS*, *PYG*, *GBE1*, *PGM*, and *HK*. In the context of glycolysis, the genes *LDH*, *PGM*, *ALDO*, *PFKA*, *NAPH^+^*, *PK*, and *TPI* were identified. The specific computational findings are outlined in **Appendix C**.

The key parameters for central network analysis were determined by the MCODE algorithm. Subsequent analysis revealed the significance of *PP2C*, *ZEP*, *ABA2*, *CYP707A*, and *GA2ox* in the ABA and GAs-related pathway, as illustrated in **Appendix Figure 8**. Similarly, *SUS*, *scrk*, *PGM*, *alpha AMY*, *HK*, *ostB*, *GBE1*, *INV*, and *PYG* were identified as central network genes in the starch and sucrose metabolism pathway. In glycolysis, the genes *HK*, *PGM*, *NAPH^+^*, *PFKA*, *LDH*, *PK*, *ALDO*, and *TPI* were identified as central networks.

By examining DEGs functions, pre- and post-germination FC, gene correlations, and the central networks extraction, key genes from various pathways were identified. Subsequent analysis revealed key genes involved in the ABA and GAs pathways, including *CYP707A* (AUR62001756), *ABA2* (AUR62021168), *GA2ox_1* (AUR62024597), and *GA2ox_2* (AUR62011753). In the pathway of starch and sucrose metabolism, pivotal genes were identified, including *INV_1* (AUR62009834), *INV_2* (AUR62039932), *malZ_1* (AUR62021097), *beta glu_6* (AUR62029347), *EGLU_1* (AUR62000202), *EGLU_2* (AUR62006550), *scrk_1* (AUR62002325), *HK* (AUR62031934), and *alpha AMY* (AUR62012986). Similarly, essential genes involved in glycolysis were *HK* (AUR62031934), *ACSS_1* (AUR62032527), and *ACSS_2* (AUR62017459).

### 3.10 RT-qPCR validation

**Figure 13:**
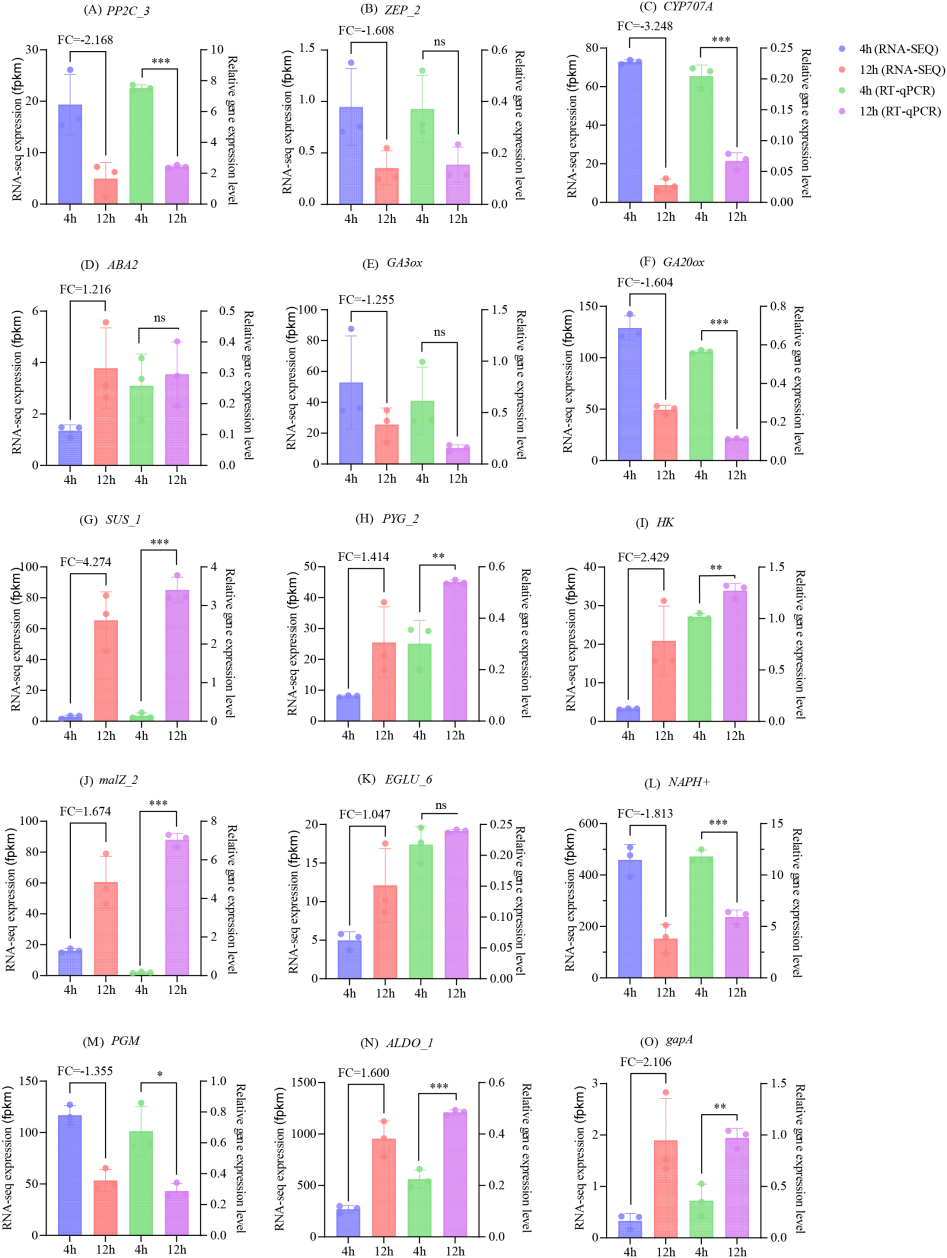
Relative expression levels of 15 genes FPKM and RT-qPCR. **(A–O)** represents *PP2C3*, *ZEP*_2, *CYP707A*, et al, as shown in the figure. "*", "**" means significant at p<0.01, while "***" means extremely significant at p<0.05. ns means "not significant".

Based on the results of Cytohubba calculations, gene correlation analysis, gene variation multiples, and a thorough literature review, a total of 15 DEGs were chosen for further validation through RT-qPCR analysis in pathways associated with diverse biological processes, with the primer sequences detailed in **Appendix D**. (**Figure 13**). The results demonstrated that the expression trends observed in the RNA-seq results were consistent with the calculated expression levels obtained from RT-qPCR.

## 4 Discussion

### 4.1 Excessive consumption of compounds during seed germination

Upon contact with water, seed germination entails the utilization of reserves and the provision of energy, which leads to the prompt initiation of internal physiological mechanisms and signal transmission (Li et al., 2022; Han and Yang, 2015). In this study, the seeds demonstrated substantial water absorption within the first 4 hours, accompanied by a rapid increase in total amylase activity that reached its maximum value. The germination of CL-2 was accompanied by a significant decrease in starch content, which was attributed to enzymatic hydrolysis catalyzed by amylase, leading to the formation of soluble sugars like maltose and glucose. Starch, as the main internal component in plants, acts as a key energy store and is crucial for various physiological processes necessary for plant growth and development (MacNeill et al., 2017).

In the process of germination, there was a notable elevation in maltose and glucose content, along with an increase in soluble sugars, while the alteration in fructose content was relatively minor. In quinoa, a study had indicated a consistent reduction in starch levels throughout seed germination, accompanied by elevated levels of fructose and glucose compared to dry seeds, followed by a subsequent decrease after an initial surge (Hao et al., 2022). Similarly, during the *in vitro* germination of *Dendrocalumus brandisii*, there was a continuous decrease in starch content, coupled with an increase in soluble sugars (Lv et al., 2021). In our study, a consistent decline in sucrose content was observed during the germination, with a minor increase noted in the later stages, showing similar findings on quinoa before (Hao et al., 2022).

Changes in content are closely associated with gene regulatory processes. Transcript analysis indicated the genes *SUS*, *INV*, and *malZ*, which facilitate the conversion of sucrose into fructose and glucose, showing differing levels of upregulation at 4 hours to 12 hours. By utilizing a combination of FC and gene correlation analysis, it was determined that *INV_1*, *INV_2*, and *malZ_1* play a crucial role in the breakdown of sucrose content during the 4 hours to 12 hours germination process. Within the pathway of starch and sucrose metabolism, *beta glu*, *EGLU*, *otsB*, *TPS*, *TREH*, and *GN* act as intermediaries in glucose production, with *beta glu_6*, *EGLU_1*, and *EGLU_2* identified as key genes in glucose synthesis during CL-2 germination.

In the pathway of fructose metabolism, *HK* and *scrk_1* have been identified as key contributors, and their upregulation plays a significant role in promoting fructose metabolism. Additionally, the limited fluctuations in fructose levels during germination may be linked to the continuous degradation of sucrose. The upregulation of amylase genes, specifically *alpha AMY* and *beta AMY*, results in a rise in maltose levels and a corresponding decrease in starch levels. Under the guidance of the *ISA*, *alpha AMY* and *beta AMY* facilitate the breakdown of starch into maltodextrin and dextrin, with maltodextrin subsequently converting into maltose through the action of amylase.

### 4.2 Enhanced sugar metabolism during seed germination

Most genes within the EMP pathway demonstrated marked upregulation, particularly those responsible for the key rate-limiting steps in EMP pathway, encoding hexokinase (*HK*), phosphofructokinase (*PFKA_1, PFKA_2, PFKA_3*), and pyruvate kinase (*PK_1, PK_2, PK_3*). Furthermore, the TCA cycle also exhibited significant gene enrichment, with a total of 10 DEGs identified in the cycle. Particularly noteworthy were the upregulation of key genes responsible for the three rate-limiting reactions of the TCA cycle, including citrate synthase genes *ACLY_1* (AUR62039280), *ACLY_2* (AUR62014075), and *ACLY_3* (AUR62005413); isocitrate dehydrogenase gene *IDH* (AUR62018235); and α-ketoglutarate dehydrogenase genes *OGDH_1* (AUR62029475) and *OGDH_2* (AUR62003361).

The citrate synthase gene family displayed the largest FC, with *ACLY_1* exhibiting a FC of 2.98, *ACLY_3* with a FC of 3.00, and *ACLY_2* with a FC of 1.71. In addition to the EMP pathway and the TCA cycle, other sugar metabolism pathways such as the pentose phosphate pathway did not show significant differences during CL-2 seed germination. In conclusion, the energy necessary for seed germination is supplied through sugar metabolism, specifically via the EMP pathway, pyruvate oxidation, and the TCA cycle.

### 4.3 ABA accumulation during seed germination under enzyme action

Based on the findings of the analysis of ABA and its associated enzymes activities, it was noted that the activities of ABA synthetic enzymes ZEP, NCED, and AAO exhibited a notable increase following seed germination, aligning with the observed fluctuations in ABA content. Further examination through correlation analysis indicated a statistically positive relationship (**Appendix E**).

The findings indicated no consistent correlation between the transcriptional levels of genes encoding ZEP, NCED, AAO, and ABA8’-H. According to the central dogma of molecular biology (Crick, 1970), gene expression involves a sequential process of DNA transcription, mRNA translation, and protein synthesis. Previous research (Gygi et al., 1999; Chick et al., 2016) has shown that various post-transcriptional mechanisms, such as processing, degradation, translation, and modifications, are essential for the activation of transcripts into functional proteins. Although transcriptional abundance can be indicative of protein expression levels, the correlation between the two is not always definitive. Post-translational modifications (PTMs) of protein are widely acknowledged as significant factors that impact protein accumulation and functionality. These modifications, such as methylation, ubiquitination, acetylation, sumoylation, persulfidation, as well as phosphorylation and dephosphorylation (Li et al., 2023; Takahashi et al., 2017; Minkoff et al., 2015; Aziz et al., 2022; Cui et al., 2023), play crucial roles in regulating protein activity. Studies on cucumber (Liu et al., 2023) and plant immunity (Zhang and Zeng, 2020) has indicated that variations in post-translational modification states can result in discrepancies between protein and transcriptional levels due to post-translational ubiquitination modifications.

Various modification methods, such as sumoylation and phosphorylation, have been reported in the regulation of phytohormones, with *SnRK2.6* being influenced by these modifications (Li et al., 2023). Simultaneously, the biological activity of Abscisic acid-insensitive 5 (*ABI5*) relies on phosphorylation and ubiquitination modifications (Yu et al., 2015; Brocard et al., 2002). In addition to the ABA signaling pathway, various phytohormones, including *DELLA* in GAs (McGinnis et al., 2003), *AHP6* in cytokinin (Mähönen et al., 2006), *CTR1* in ethylene (Kieber et al., 1993), and *BIN2* in Brassinosteroids(BR) (He et al., 2002), participate in PTMs that regulate their bioactivity. Hence, it was deduced that the discrepancy between the transcriptional regulation of target genes and enzyme activity within the ABA biosynthesis pathway could potentially be influenced by biological mechanisms such as transcriptional processing and PTMs.

The content of ABA is a result of the combined effects of multiple genes (Song et al., 2016; Raghavendra et al., 2010). Notably, DEGs including *ZEP*, *LUT5*, *ABA2*, and *CYP707A* were identified within the ABA biosynthetic pathway. Specifically, the results of this study indicated a significant upregulation of *ABA2* and a marked downregulation of *CYP707A*. It can be inferred that the accumulation of ABA between 4 and 12 hours is primarily regulated by the coordinated activity of *ABA2* and *CYP707A*. Furthermore, the expression profiles of ABA-related genes identified in this research align with previous white quinoa (BL) sequencing data (RNA seq data available at http://trace.ncbi.nlm.nih.gov/Traces/sra, PRJNA590581) (Wu et al., 2020).

In the hormone signaling pathways, *PYL*, *SnRK2*, and *ABF* were identified as upregulated genes, while *PP2C* expression was downregulated, resulting in negative regulation of seed germination. The observed gene expression pattern in BL (Wu et al., 2020)was found to be consistent with that of CL-2 in this study. Our previous research also found that CL-2 variety is insensitive to endogenous ABA (Zeng et al., 2024). Meanwhile, there may be differences in the sensitivity of different quinoa varieties to ABA, although we have not conducted in-depth research on this.

Despite the traditional understanding of ABA as a suppressor of seed germination (Ali et al., 2022), previous studies on quinoa have showed that seeds with lower dormancy levels have higher endogenous ABA levels (Ceccato et al., 2015). This indicates that the endogenous ABA content in quinoa may not be directly associated with dormancy. Moreover, diverse cultivars of quinoa demonstrate differential responses to ABA at different stages, with seeds exhibiting reduced sensitivity to ABA during dormancy release and heightened sensitivity to GAs signaling (McGinty et al., 2021). Additionally, researchers (Ceccato et al., 2015) suggested notable disparities in the quinoa germination efficiency attributed to variations in sowing timing and seed coat thickness. Similarly, analysis of 189 quinoa materials using phenotype modeling indicated that 141 of the materials did not exhibit primary dormancy, and revealed a correlation between seed coat thickness, eccentricity, and quinoa dormancy and germination (McGinty et al., 2023). Overall, the growing body of research on quinoa indicates that its germination traits are impacted by various factors, with ABA content potentially not playing a direct role.

### 4.4 GA_3_ plays a positive regulatory role in seed germination process

GA_3_ has been identified as a crucial phytohormone involved in the regulation of seed germination, root and stem elongation, and cell elongation (Ritonga et al., 2023). The rapid uptake of water leads to an increase in GA_3_ levels, indicating a strong relationship between seed moisture content and GA_3_ levels. Following-germination, there is a notable rise in GA_3_ content in quinoa seeds, similar to the process observed in the release of dormancy wheat (*Triticum aestivum* L.) seeds (Wang et al., 2022), Japanese apricot seeds (*Prunus mumeSieb*. et Zucc) (Kita et al., 2007),and tomato seeds (Zhang et al., 2022). Notably, in quinoa seeds, there is a rapid increase in GA_3_ levels within the first 4 hours of germination, followed by a decrease at the 4-hour mark, and subsequently, a sustained minor fluctuation in GA_3_ levels from 8 hours to 20 hours. Previous study on cotton (*Gossypium hirsutum* L.) (Xiao et al., 2019) has demonstrated a similar pattern of GA_3_ levels peaking and subsequently declining during seed germination, whereas in grapes (Zheng et al., 2018), GA_3_ levels decrease during the transition from dormancy induction to maintenance and release. As a result, during the germination of quinoa seeds, there is a simultaneous production and utilization of endogenous GA_3_, with consumption rates being lower than synthesis rates, leading to a trend in GA_3_ levels during the process.

The enzymes GA20ox and GA3ox play key roles in converting inactive forms of GAs into active GAs, while GA2ox is responsible for GAs deactivation (Urbanová et al., 2013; Rieu et al., 2008). Analysis of enzyme activity demonstrated an increase in synthesis enzyme activity following seeds germination, promoting the accumulation of GAs. The activity of GA2ox initially increased during the early stages of germination, followed by a decline in the later stages, which suggests GA2ox plays a key role in regulating GAs homeostasis. The lack of significant correlation between GA_3_ and the activities of GA20ox, GA3ox, and GA2ox implies that this observation may be attributed to the presence of various active forms of GAs, including GA_1_, GA_3_, GA_4_, and GA_7_ (Liu et al., 2014).

The integrated transcriptional results and correlation analysis indicated that the increase in GA_3_ content at 4h compared to 12h is primarily attributed to the downregulation of *GA2ox_1* and *GA2ox_2*. Report have shown that the overexpression of *GA2ox* results in plants displaying GA-deficient phenotypes (Wuddineh et al., 2015). In maize subjected to cold and drought stress, the expression levels of *GA2ox* were found to be inversely related to GAs content (Li et al., 2021). Numerous studies have demonstrated the significant involvement of *GA2ox* in the regulation of GAs metabolism. Despite the upregulation of the upstream GAs synthesis gene *KAO*, the coordinated activity of *CYP701_1*, *CYP701_2*, *GA20ox*, *GA3ox*, *GA2ox_1*, and *GA2ox_2* results in a reduction in GA_3_ content. Notably, *GA2ox_1* and *GA2ox_2* play pivotal roles in modulating the GAs pathway.

## 5 Conclusion

The study conducted an experiment to analyze the content of endogenous physiological and biochemical indicators during different stages of quinoa seed germination. By integrating the findings from the 4-hour and 12-hour transcriptome analyses, the study investigated the dynamic changes in physiology and transcription levels. The results indicated that starch and sucrose played a crucial role in providing energy for seed germination, leading to an overall increase in levels of soluble sugars, soluble proteins, and small-molecule sugars such as glucose and maltose.

Enzymes including ZEP, NCED, and AAO play a crucial role in the regulation of ABA synthesis, resulting in an increase in endogenous ABA content during seed germination. The study suggests that the relationship between the endogenous ABA content and germination status in quinoa may not be definitive. The activity of GA20ox, GA3ox, and GA2ox enzymes collectively governs the content of GA_3_, which, in turn, facilitates seed germination. The analysis of the transcriptome revealed 3349 DEGs, with KEGG annotation indicating enhanced activity in hormone signaling, starch and sucrose metabolism, the EMP pathway, and the TCA cycle. This study investigated the mechanisms underlying quinoa germination at physiological and genetic levels, thereby laying a theoretical foundation for future investigations on quinoa seeds. Meanwhile, comprehending the intricate mechanisms underlying quinoa seed germination and ear sprouting is critically important for the cultivation and production of quinoa.

## Data Availability

The datasets presented in this study can be found in online repositories. The names of the repository/repositories and accession number(s) can be found below: https://www.ncbi.nlm.nih.gov/genbank/, PRJNA1028334.
