# Supplementary material for: Integrating transcriptomic, physiological, and biochemical studies revealing the role of endogenous ABA and GA3 in the germination of quinoa seed
Source: Front Plant Sci. 2026 Mar 12;17:1722072. doi: 10.3389/fpls.2026.1722072 (PMC13017246; doi:10.3389/fpls.2026.1722072)
Supplement: Supplementary file 1 [file DataSheet1.docx]

**Appendix A. Details of measurement methods for physiological and biochemical indicators**

**1.1 Determination of small molecule sugar conten**t

The contents of fructose, glucose, sucrose, and maltose during seed germination were determined by High Performance Liquid Chromatography. The extraction conditions were as follows: 3 mL of ultrapure water was used for thorough shaking, followed by a 30-minute low-temperature water bath with ultrasonic treatment. The mixture was then centrifuged at 13400 g for 5 minutes at 4℃. The supernatant was collected, and this process was repeated 3 times. The supernatants were combined and diluted to a final volume of 10 mL. 1 mL of the supernatant was filtered through a 0.22 μm aqueous phase filter membrane and stored at -20℃ for subsequent use. The chromatographic column used was XBridge BEH AMIDE (4.6 mm × 250 mm, 5 μm) with a column temperature of 35℃. The mobile phase consisted of a mixture of H_2_O and acetonitrile, and the flow rate was set to 1 mL min^-1^. The injection volume was 10 µL.

**1.2 Determination of ABA and GA_3_ Content**

**1.2.1 Preparation of Standard Curve Solutions**

A quantity of 988 μL of methanol solution was added to a 1.5 mL centrifuge tube, followed by the addition of 2 μL of each hormone internal standard stock solution at a concentration of 500 μg mL^-1^. The mixture was then shaken uniformly to prepare the internal standard working solution with a final concentration of 1 μg mL^-1^ for subsequent use. Methanol solutions were used to prepare standard curves with final concentrations of 0.1 ng mL^-1^, 0.2 ng mL^-1^, 0.5 ng mL^-1^, 2 ng mL^-1^, 5 ng mL^-1^, 20 ng mL^-1^, 50 ng mL^-1^, and 200 ng mL^-1^.

**1.2.2 Extraction of Plant Hormones**

The samples were rapidly ground into powder in liquid nitrogen. Exactly 1.000 g of the sample was weighed into a tube, and 10 mL of acetonitrile solution was added along with 8 μL of the internal standard stock solution. The mixture was extracted overnight at 4℃. Subsequently, the mixture was centrifuged at 13400 g for 5 minutes at 4℃, and the supernatant was collected. The precipitate was re-extracted twice with 5 mL of acetonitrile solution, and the supernatants were combined Appropriat amounts of C18 and GCB were added to purify the mixture of impurities. After centrifugation at 13400 g for 5 minutes at 4℃, the supernatant was collected and dried by nitrogen gas. The dried sample was then dissolved in 400 mL of methanol, filtered through a 0.22 μm organic phase filter membrane, and stored in a -20℃ freezer for subsequent detection on the instrument.

**1.2.3 Detection Conditions for Plant Hormones**

Liquid Chromatography Conditions: The chromatographic column used was a Poroshell 120 SB-C18 reversed-phase column (2.1 × 150, 2.7 μm). The column temperature was set at 30℃. The mobile phase consisted of a mixture of methanol with 0.1% formic acid (phase A) and water with 0.1% formic acid (phase B). The injection volume was 2 µL. An elution gradient was applied.

Table A.1 Liquid Chromatography Gradient Parameters

| Time（min） | Speed（mL/min） | A% |
| --- | --- | --- |
| 0-1 | 0.3 | 20 |
| 1-3 | 0.3 | From 20 to 50 |
| 3-9 | 0.3 | From 50 to 80 |
| 9-10.5 | 0.3 | 80 |
| 10.5-10.6 | 0.3 | From 80 to 20 |
| 10.6-13.5 | 0.3 | 20 |

Mass Spectrometry Parameters: Ionization Mode: ESI positive and negative ion modes monitored separately; Scan Type: MRM (Multiple Reaction Monitoring); Curtain Gas: 15 psi; Spray Voltage: +4500 V for positive mode, -4000 V for negative mode; Nebulizer Gas Pressure: 65 psi; Auxiliary Gas Pressure: 70 psi; Nebulizer Temperature: 400℃.

Table A.2 Selected reaction monitoring conditions for protonated or deprotonated plant hormones ([M+H] + or[M-H]-)

| Hormone | Polarity | Parent Ion  （m/z） | Daughter Ion  （m/z） | Declustering Potential  （V） | Collision Energy  （V） |
| --- | --- | --- | --- | --- | --- |
| ABA | - | 263.1 | 153.1*/204.2 | -60 | -14/-27 |
| GA_3_ | - | 345.2 | 143.0*/239.2 | -80 | -30/-33 |

**1.3 Detection of ABA and GAs-Related Enzyme Activity**

The enzyme activity measurements for ZEP, NCED, AAO, ABA8'-H, GA20ox, GA3ox, and GA2ox were conducted by ELISA kits from Jiangsu Meimian Industrial Co., Ltd. The specific steps were as follows: reagents, samples, and standards were prepared; samples and standards were added; they were incubated at 37℃ for 30 minutes; the plates were washed 5 times; enzyme-labeled reagents were added; they were incubated at 37℃ for 30 minutes, again; the plates were washed 5 more times and chromogenic solution was added; they were incubated at 37℃ for 10 minutes for color development; after color development, stop solution was added; the OD value was read within 15 minutes; and the target enzyme activity was calculated.

**Appendix Table 1.** Sequence information after quality control

| Sample ID | Before QC | After QC | Q20 rate | Q30 rate | GC content | Overall mapping rate |
| --- | --- | --- | --- | --- | --- | --- |
| 4h-01 | 6.78G | 6.47G | 97.71% | 93.12% | 44.78% | 97.50% |
| 4h-02 | 6.42G | 6.12G | 97.57% | 92.86% | 44.48% | 97.40% |
| 4h-03 | 6.6G | 6.27G | 97.38% | 92.44% | 44.85% | 97.20% |
| 12h-01 | 6.32G | 6.04G | 97.55% | 92.80% | 43.69% | 97.40% |
| 12h-02 | 6.35G | 6.07G | 97.61% | 92.96% | 44.07% | 96.70% |
| 12h-03 | 6.43G | 6.13G | 97.47% | 92.62% | 44.07% | 97.20% |

**Appendix B. Detailed information for full name and ID of genes**

Table B.1 Gene description

| Name | Gene ID | Description |
| --- | --- | --- |
| *ZEP_1* | *AUR62003884* | Zeaxanthin Epoxidase |
| *ZEP_2* | *AUR62001926* | Zeaxanthin Epoxidase |
| *CYP707A* | *AUR62001756* | (+)-Abscisic Acid 8'-Hydroxylase |
| *ABA2* | *AUR62021168* | Xanthoxin Dehydrogenase |
| *LUT5* | *AUR62006725* | Beta-Ring Hydroxylase |
| *KAO* | *AUR62023338* | ent-Kaurenoic Acid Monooxygenase |
| *CYP701_1* | *AUR62035923* | ent-Kaurene Oxidase |
| *GA20ox* | *AUR62021271* | Gibberellin-44 Dioxygenase |
| *GA2ox_1* | *AUR62024597* | Gibberellin 2Beta-Dioxygenase |
| *GA2ox_2* | *AUR62011753* | Gibberellin 2Beta-Dioxygenase |
| *CYP701_2* | *AUR62035325* | ent-Kaurene Oxidase |
| *GA3ox* | *AUR62016415* | Gibberellin 3Beta-Dioxygenase |
| *ABF_1* | *AUR62009766* | ABA Responsive Element Binding Factor |
| *ABF_2* | *AUR62009948* | ABA Responsive Element Binding Factor |
| *ABF_3* | *AUR62001422* | ABA Responsive Element Binding Factor |
| *ABF_4* | *AUR62039213* | ABA Responsive Element Binding Factor |
| *ABF_5* | *AUR62018468* | ABA Responsive Element Binding Factor |
| *PYL_1* | *AUR62044383* | Abscisic Acid Receptor Pyr/Pyl Family |
| *PYL_2* | *AUR62022950* | Abscisic Acid Receptor Pyr/Pyl Family |
| *PYL_3* | *AUR62004215* | Abscisic Acid Receptor Pyr/Pyl Family |
| *PP2C_1* | *AUR62006130* | Protein Phosphatase 2C |
| *PP2C_2* | *AUR62040699* | Protein Phosphatase 2C |
| *PP2C_3* | *AUR62020044* | Protein Phosphatase 2C |
| *PP2C_4* | *AUR62001731* | Protein Phosphatase 2C |

Continued

| Name | Gene ID | Description | |
| --- | --- | --- | --- |
| *SnRK2_1* | *AUR62003254* | Serine/Threonine-Protein Kinase Srk2 | |
| *SnRK2_2* | *AUR62027216* | Serine/Threonine-Protein Kinase Srk2 | |
| *GID1_1* | *AUR62000577* | Gibberellin Receptor Gid1 | |
| *GID1_2* | *AUR62006931* | Gibberellin Receptor Gid1 | |
| *otsB_1* | *AUR62018916* | Trehalose 5-Phosphate Phosphatase | |
| *otsB_2* | *AUR62002194* | Trehalose 6-Phosphate Phosphatase | |
| *INV_1* | *AUR62009834* | Beta-Fructofuranosidase | |
| *INV_2* | *AUR62039932* | Beta-Fructofuranosidase |  |
| *INV_3* | *AUR62007474* | Beta-Fructofuranosidase |  |
| *INV_4* | *AUR62044189* | Beta-Fructofuranosidase |  |
| *malZ_1* | *AUR62021097* | Alpha-Glucosidase |  |
| *malZ_2* | *AUR62031441* | Alpha-Glucosidase |  |
| *SUS_1* | *AUR62025310* | Sucrose Synthase |  |
| *SUS_2* | *AUR62028315* | Sucrose Synthase |  |
| *SUS_3* | *AUR62008699* | Sucrose Synthase |  |
| *SUS_4* | *AUR62025532* | Sucrose Synthase |  |
| *HK* | *AUR62031934* | Hexokinase |  |
| *scrK_1* | *AUR62002325* | Fructokinase |  |
| *scrK_2* | *AUR62023862* | Fructokinase |  |
| *Beta glu_1* | *AUR62007525* | Beta-Glucosidase |  |
| *Beta glu_2* | *AUR62003117* | Beta-Glucosidase |  |
| *GN_1* | *AUR62021934* | Glucan Endo-1,3-Beta-Glucosidase 1/2/3 |  |
| *GN_2* | *AUR62018216* | Glucan Endo-1,3-Beta-Glucosidase 1/2/4 |  |
| *GN_3* | *AUR62043470* | Glucan Endo-1,3-Beta-Glucosidase 1/2/5 |  |
| *GN_4* | *AUR62014160* | Glucan Endo-1,3-Beta-Glucosidase 1/2/6 |  |
| *GN_5* | *AUR62020748* | Glucan Endo-1,3-Beta-Glucosidase 5/6 |  |

Continued

| Name | Gene ID | Description |
| --- | --- | --- |
| *Beta glu_3* | *AUR62039730* | Beta-Glucosidase |
| *Beta glu_4* | *AUR62036281* | Beta-Glucosidase |
| *Beta glu_5* | *AUR62014289* | Beta-Glucosidase |
| *Beta glu_6* | *AUR62029347* | Beta-Glucosidase |
| *Beta glu_7* | *AUR62021986* | Beta-Glucosidase |
| *Beta glu_8* | *AUR62009820* | Beta-Glucosidase |
| *TPS_1* | *AUR62013953* | Trehalose 6-Phosphate Synthase/Phosphatase |
| *TPS_2* | *AUR62018691* | Trehalose 7-Phosphate Synthase/Phosphatase |
| *TPS_3* | *AUR62008239* | Trehalose 8-Phosphate Synthase/Phosphatase |
| *TREH* | *AUR62019888* | Alpha,Alpha-Trehalase |
| *GN_6* | *AUR62027170* | Glucan Endo-1,3-Beta-Glucosidase 5/6 |
| *ENPP_1* | *AUR62027493* | Ectonucleotide Pyrophosphatase/Phosphodiesterase Family Member 1/3 |
| *ENPP_2* | *AUR62033988* | Ectonucleotide Pyrophosphatase/Phosphodiesterase Family Member 1/4 |
| *PGM* | *AUR62021270* | Phosphoglucomutase |
| *glgC_1* | *AUR62037153* | Glucose-1-Phosphate Adenylyltransferase |
| *glgC_2* | *AUR62016760* | Glucose-2-Phosphate Adenylyltransferase |
| *GBE1* | *AUR62022516* | 1,4-Alpha-Glucan Branching Enzyme |
| *glgA_1* | *AUR62044272* | Starch Synthase |
| *glgA_2* | *AUR62021943* | Starch Synthase |
| *glgA_3* | *AUR62006085* | Starch Synthase |
| *PYG_1* | *AUR62018166* | Glycogen Phosphorylase |
| *PYG_2* | *AUR62001699* | Glycogen Phosphorylase |
| *PYG_3* | *AUR62007939* | Glycogen Phosphorylase |
| *PYG_4* | *AUR62003120* | Glycogen Phosphorylase |

Continued

| Name | Gene ID | Description |
| --- | --- | --- |
| *ISA* | *AUR62027816* | Isoamylase |
| *Beta AMY* | *AUR62007199* | Beta-Amylase |
| *Alpha AMY* | *AUR62012986* | Alpha-Amylase |
| *EGLU_1* | *AUR62000202* | Endoglucanase |
| *EGLU_2* | *AUR62006550* | Endoglucanase |
| *EGLU_3* | *AUR62024228* | Endoglucanase |
| *EGLU_4* | *AUR62016821* | Endoglucanase |
| *EGLU_5* | *AUR62025533* | Endoglucanase |
| *EGLU_6* | *AUR62033840* | Endoglucanase |
| *EGLU_7* | *AUR62002187* | Endoglucanase |
| *EGLU_8* | *AUR62020286* | Endoglucanase |
| *PGM* | *AUR62021270* | Phosphoglucomutase |
| *HK* | *AUR62031934* | Hexokinase |
| *GALM* | *AUR62031614* | Aldose 1-Epimerase |
| *E5.1.3.15* | *AUR62019795* | Glucose-6-Phosphate 1-Epimerase |
| *PFP* | *AUR62021897* | Diphosphate-Dependent Phosphofructokinase |
| *PFKA_1* | *AUR62031952* | 6-Phosphofructokinase 1 |
| *PFKA_2* | *AUR62033274* | 6-Phosphofructokinase 1 |
| *PFKA_3* | *AUR62039818* | 6-Phosphofructokinase 1 |
| *PFKA_4* | *AUR62005870* | 6-Phosphofructokinase 1 |
| *ALDO_1* | *AUR62041432* | Fructose-Bisphosphate Aldolase, Class I |
| *ALDO_2* | *AUR62033562* | Fructose-Bisphosphate Aldolase, Class I |
| *ALDO_3* | *AUR62033531* | Fructose-Bisphosphate Aldolase, Class I |
| *TPI* | *AUR62039374* | Triosephosphate Isomerase (Tim) |
| *gapA* | *AUR62005566* | Glyceraldehyde 3-Phosphate Dehydrogenase (Phosphorylating) |
| *MINPP1* | *AUR62022935* | Multiple Inositol-Polyphosphate Phosphatase |

Continued

| Name | Gene ID | Description |
| --- | --- | --- |
| *ATP_1* | *AUR62032871* | Phosphoenolpyruvate Carboxykinase (Atp) |
| *ATP_2* | *AUR62005878* | Phosphoenolpyruvate Carboxykinase (Atp) |
| *PK_1* | *AUR62031385* | Pyruvate Kinase |
| *PK_2* | *AUR62031459* | Pyruvate Kinase |
| *PK_3* | *AUR62021072* | Pyruvate Kinase |
| *LDH* | *AUR62007499* | L-Lactate Dehydrogenase |
| *PDHB_1* | *AUR62002731* | Pyruvate Dehydrogenase E1 Component Subunit Beta |
| *PDHB_2* | *AUR62022407* | Pyruvate Dehydrogenase E2 Component Subunit Beta |
| *NAPH+_1* | *AUR62006286* | Alcohol Dehydrogenase (Nadp+) |
| *NAPH+_2* | *AUR62006284* | Alcohol Dehydrogenase (Nadp+) |
| *NAPH+_3* | *AUR62026333* | Alcohol Dehydrogenase (Nadp+) |
| *NAPH+_4* | *AUR62026335* | Alcohol Dehydrogenase (Nadp+) |
| *NAPH+_5* | *AUR62016843* | Alcohol Dehydrogenase (Nadp+) |
| *ADH5_1* | *AUR62036905* | Alcohol Dehydrogenase |
| *ADH5_2* | *AUR62013830* | Alcohol Dehydrogenase |
| *ACSS_1* | *AUR62032527* | Acetyl-Coa Synthetase |
| *ACSS_2* | *AUR62017459* | Acetyl-Coa Synthetase |
| *ALDH_1* | *AUR62000539* | Aldehyde Dehydrogenase (Nad+) |
| *ALDH_2* | *AUR62000112* | Aldehyde Dehydrogenase (Nad+) |
| *MDH2* | *AUR62014203* | Malate Dehydrogenase |
| *IDH* | *AUR62018235* | Isocitrate Dehydrogenase |
| *OGDH_1* | *AUR62029475* | 2-Oxoglutarate Dehydrogenase E1 Component |
| *OGDH_2* | *AUR62003361* | 2-Oxoglutarate Dehydrogenase E1 Component |
| *SDHB_1* | *AUR62003783* | Succinate Dehydrogenase (Ubiquinone) Iron-Sulfur Subunit |
| *SDHB_2* | *AUR62002018* | Succinate Dehydrogenase (Ubiquinone) Iron-Sulfur Subunit |
| *SDHB_3* | *AUR62006089* | Succinate Dehydrogenase (Ubiquinone) Iron-Sulfur Subunit |

Continued

| Name | Gene ID | Description |
| --- | --- | --- |
| *ACLY_1* | *AUR62039280* | ATP Citrate (Pro-S)-Lyase |
| *ACLY_2* | *AUR62014075* | ATP Citrate (Pro-S)-Lyase |
| *ACLY_3* | *AUR62005413* | ATP Citrate (Pro-S)-Lyase |


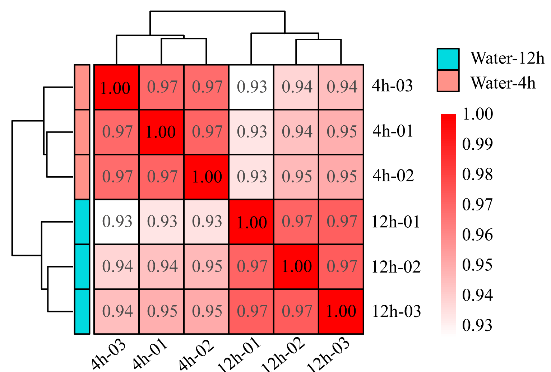


**Appendix Figure 1.** Spearman correlation heatmap between expression level samples.


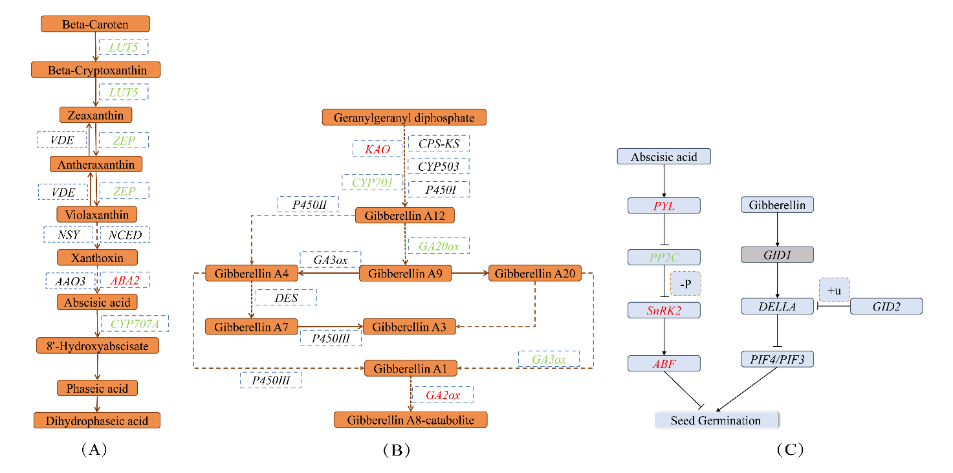
**Appendix Figure 2.** ABA, GAs synthesis and signal transduction pathways. A, B, and C represent the ABA synthesis metabolic pathway, GAs synthesis metabolic pathway, and ABA and GAs-related signaling pathway, respectively. The orange box in the figure represents the compound product, and the dashed box represents the gene involved in this step. The green font represents downregulation, the red font represents upregulation, and the black font represents the gene is not differentially expressed. The gray box filled with black font indicates that among the multiple homologous genes contained in this gene, there are both upregulated and downregulated genes. The dashed line signifies that this step encompasses multiple subprocesses.


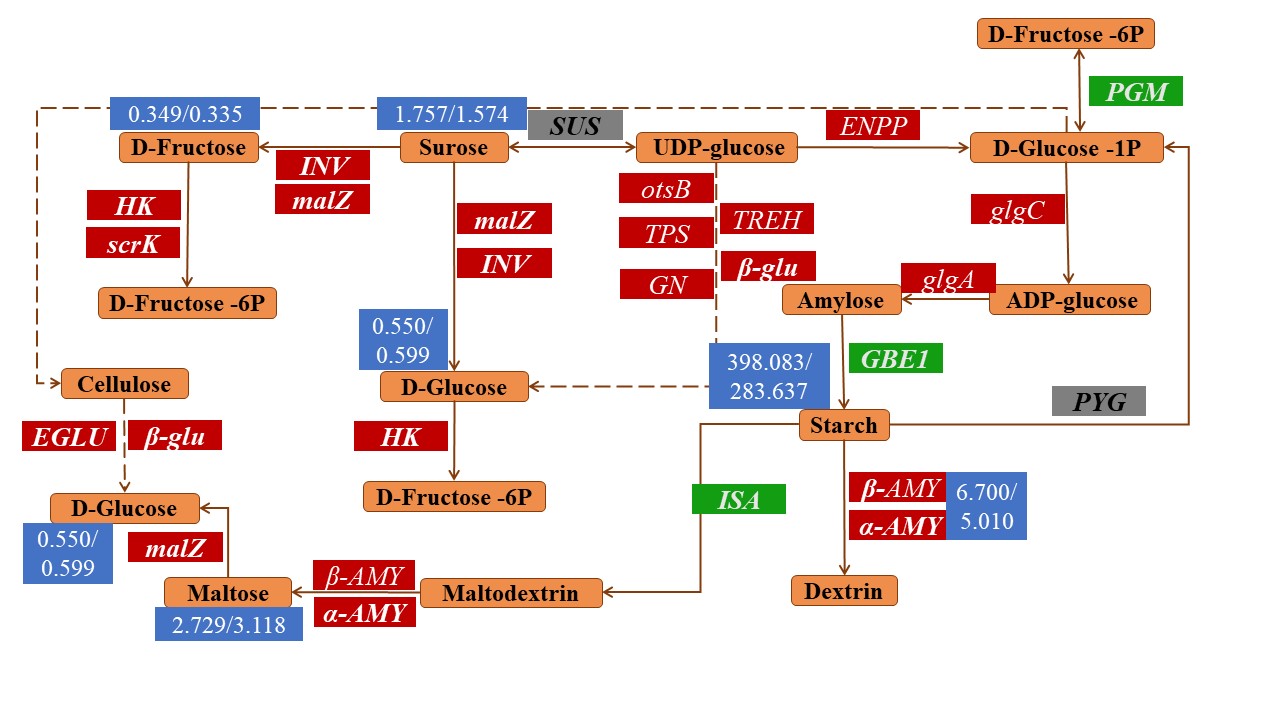


**Appendix Figure 3.** Metabolic pathways of starch and sucrose. The orange box in the figure represents the compound product, and the dashed box represents the gene involved in this step. The green font represents downregulation，the red font represents upregulation. The gray box filled with black font indicates that among the multiple homologous genes contained in this gene, there are both upregulated and downregulated genes. The blue box and white font indicate the content of the product at 4 h and 12 h (4 h/12 h), The dashed line signifies that this step encompasses multiple subprocesses.


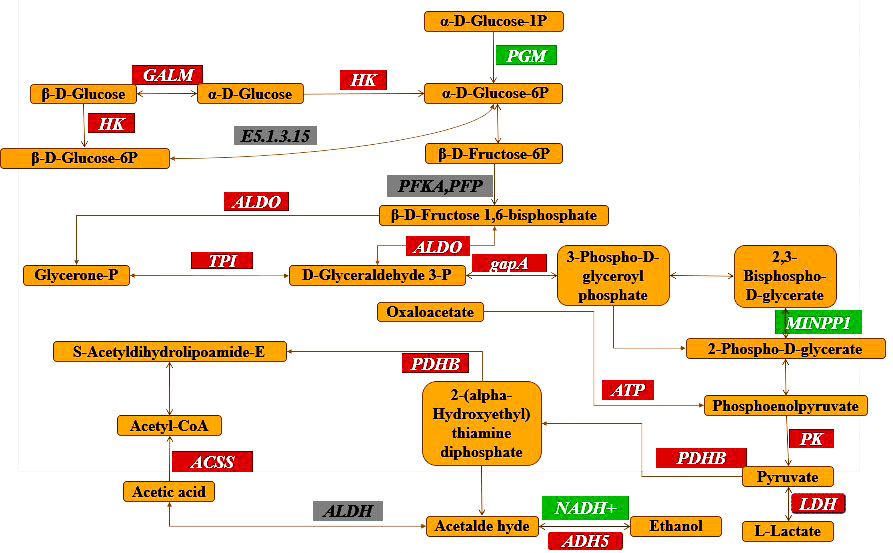


**Appendix Figure 4**. Glycolysis Pathway. The orange box in the figure represents the compound product, and the dashed box represents the gene involved in this step. The green font represents downregulation，the red font represents upregulation. The gray box filled with black font indicates that among the multiple homologous genes contained in this gene, there are both upregulated and downregulated genes. The dashed line signifies that this step encompasses multiple subprocesses.


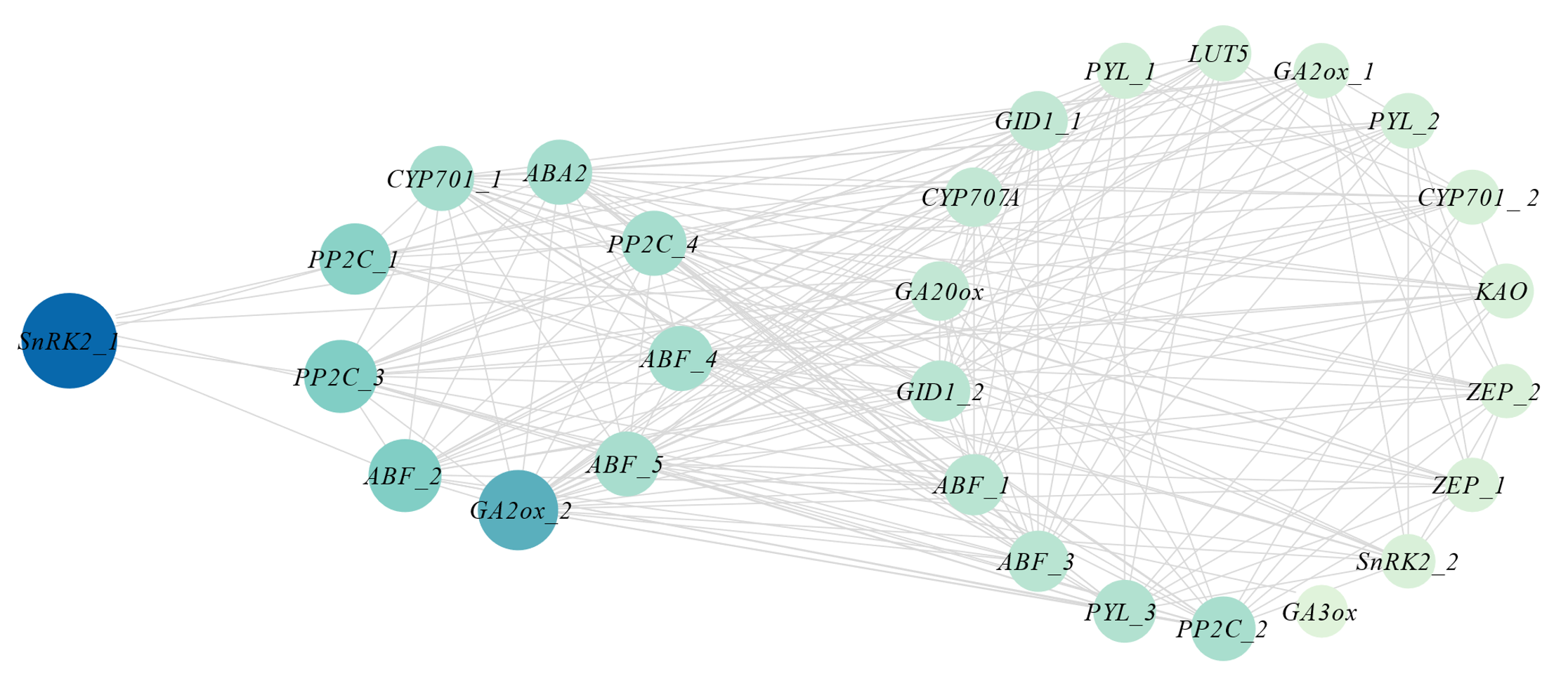


**Appendix Figure 5**. Correlation of differentially expressed genes in ABA and GAs synthesis metabolism.


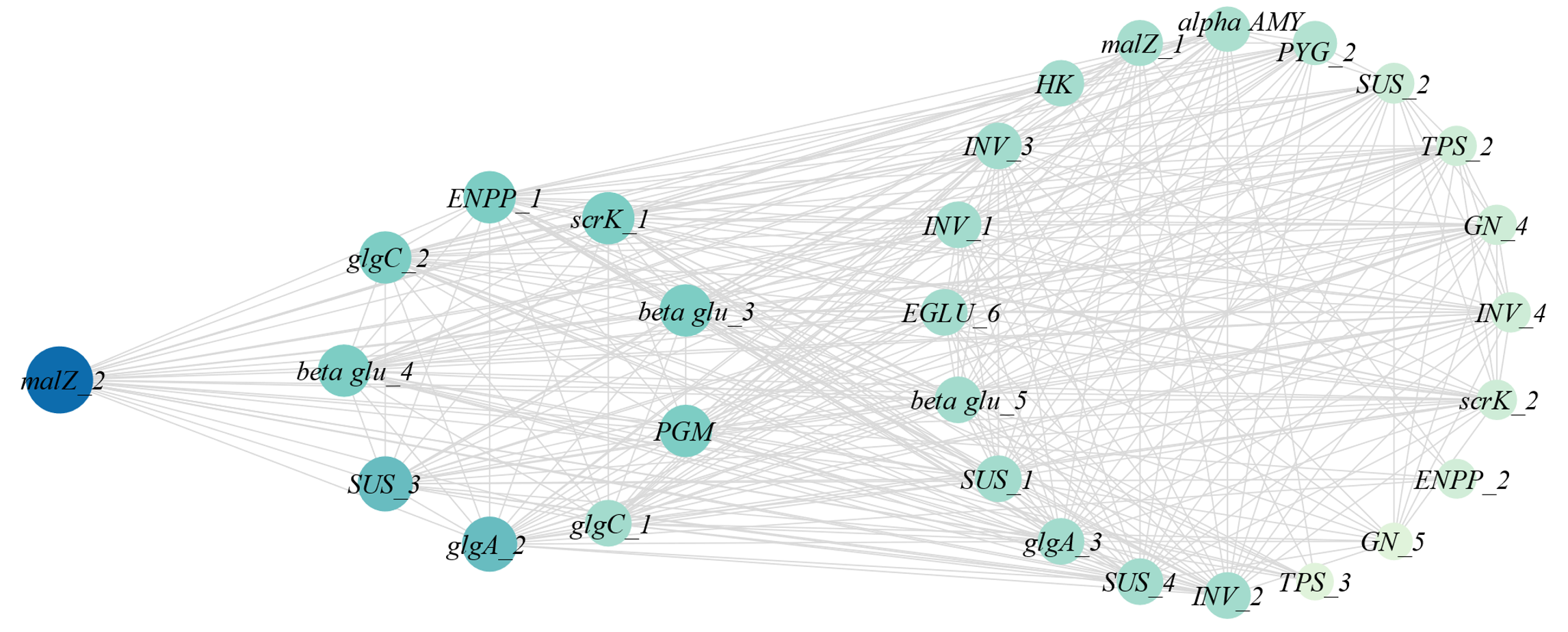


**Appendix Figure 6**. Gene correlation of starch and sucrose metabolism differences.


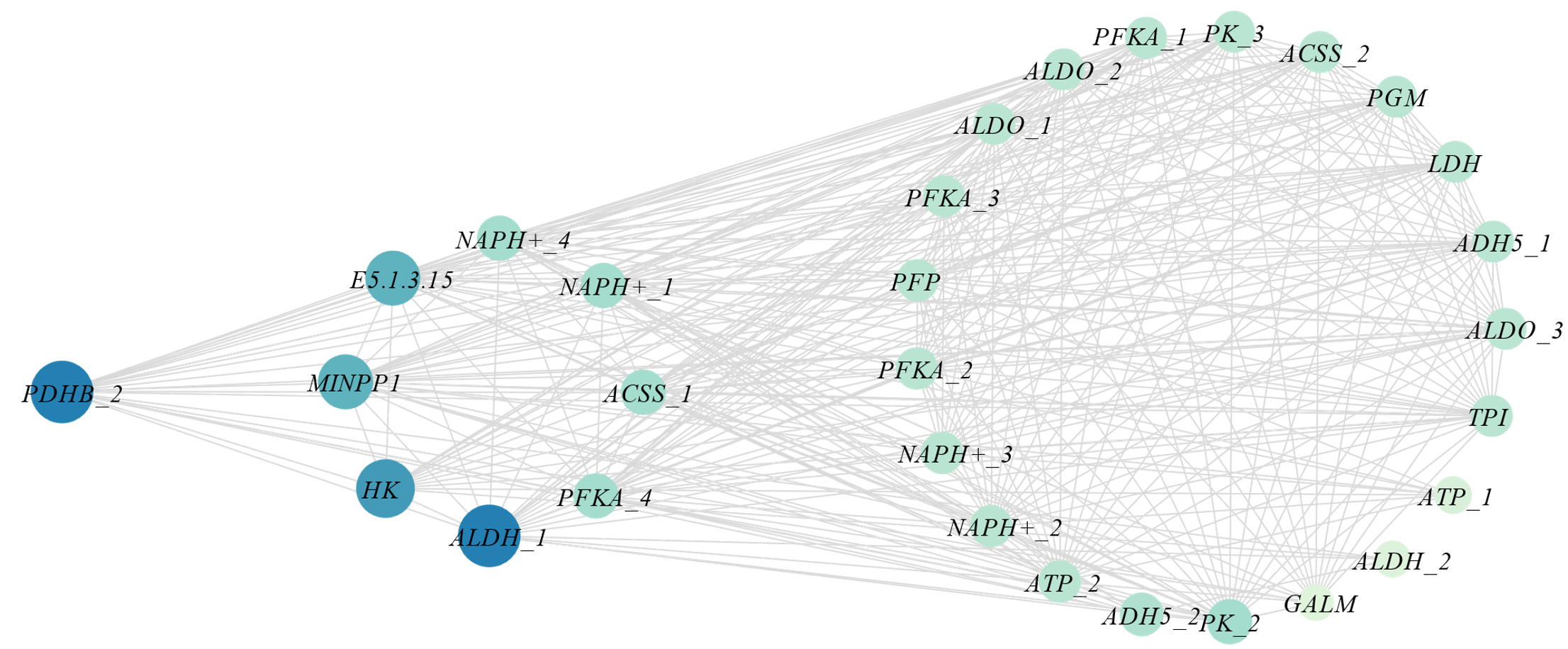


**Appendix Figure 7.** Correlation of differentially expressed genes in sugar metabolism.


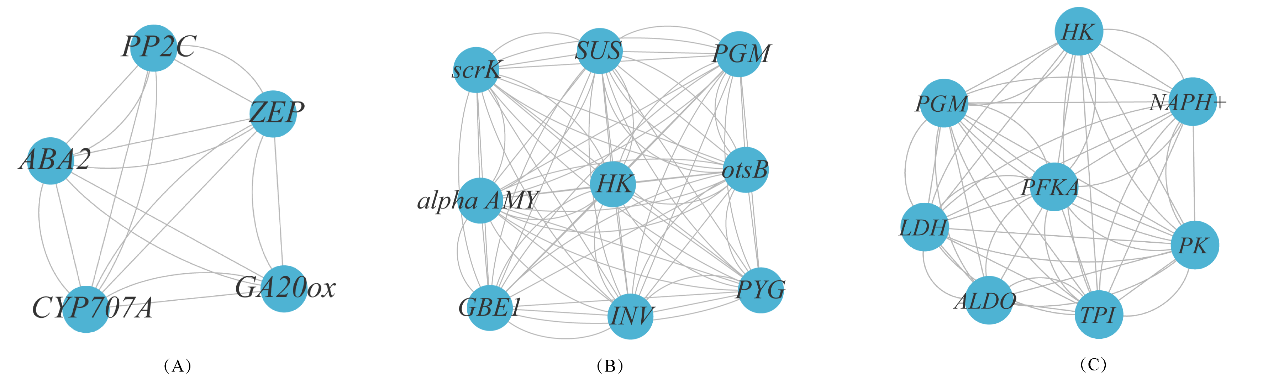


**Appendix Figure 8.** Network diagrams of different path centers. A, B, and C represent ABA, GA metabolism and signaling pathways, starch and sucrose metabolism pathways, and glycolysis pathways, respectively.

**Appendix C.** **Cytohubba coefficient calculation results**

Table C.1 The calculation result of ABA and GAs related gene of Cytohubba

| Rank | MCC | | MNC | | Betweenness | | Stress | | Closeness | | Degree | | DMNC | | EPC | | Radiality | |
| --- | --- | --- | --- | --- | --- | --- | --- | --- | --- | --- | --- | --- | --- | --- | --- | --- | --- | --- |
|  | Name | Score | Name | Score | Name | Score | Name | Score | Name | Score | Name | Score | Name | Score | Name | Score | Name | Score |
| 1 | *GA3ox* | 62 | *GA3ox* | 7 | *ABA2* | 6.40 | *ABA2* | 136 | *GA3ox* | 7.50 | *GA3ox* | 14 | *CYP707A* | 1.05 | *GA3ox* | 4.461 | *GA3ox* | 3.38 |
| 2 | *CYP707A* | 54 | *GA20ox* | 6 | *ZEP* | 6.40 | *ZEP* | 136 | *GA20ox* | 7.00 | *GA20ox* | 12 | *GA3ox* | 1.02 | *CYP707A* | 4.251 | *ZEP* | 3.25 |
| 3 | *ABA2* | 50 | *ABA2* | 6 | *GA3ox* | 5.57 | *GA3ox* | 120 | *ABA2* | 7.00 | *ABA2* | 12 | *GA20ox* | 0.95 | *GA20ox* | 4.215 | *GA20ox* | 3.25 |
| 4 | *ZEP* | 50 | *CYP707A* | 6 | *GA20ox* | 4.50 | *GA20ox* | 104 | *CYP707A* | 7.00 | *CYP707A* | 12 | *ABA2* | 0.95 | *ABA2* | 4.211 | *CYP707A* | 3.25 |
| 5 | *GA20ox* | 36 | *ZEP* | 6 | *CYP707A* | 3.07 | *CYP707A* | 64 | *ZEP* | 7.00 | *ZEP* | 12 | *ZEP* | 0.95 | *ZEP* | 4.136 | *ABA2* | 3.25 |

Table C.2 The calculation result of genes related to starch and sucrose metabolism pathway by Cytohubba

| Rank | MCC | | MNC | | Betweenness | | Stress | | Closeness | | Degree | | DMNC | | EPC | | Radiality | |
| --- | --- | --- | --- | --- | --- | --- | --- | --- | --- | --- | --- | --- | --- | --- | --- | --- | --- | --- |
|  | Name | Score | Name | Score | Name | Score | Name | Score | Name | Score | Name | Score | Name | Score | Name | Score | Name | Score |
| 1 | *GBE1* | 528 | *SUS* | 12 | *INV* | 113.00 | *INV* | 2328 | *SUS* | 14.83 | *otsB* | 34 | *scrK* | 1.75 | *otsB* | 9.41 | *SUS* | 3.89 |
| 2 | *PYG* | 528 | *otsB* | 12 | *Beta glu* | 35.00 | *Beta glu* | 960 | *GBE1* | 14.83 | *SUS* | 26 | *INV* | 1.75 | *SUS* | 9.223 | *Alpha AMY* | 3.89 |
| 3 | *ISA* | 504 | *GBE1* | 12 | *otsB* | 27.36 | *otsB* | 824 | *PYG* | 14.83 | *Alpha AMY* | 26 | *HK* | 1.58 | *Alpha AMY* | 9.137 | *INV* | 3.89 |
| 4 | *PGM* | 480 | *PYG* | 12 | *Alpha AMY* | 21.83 | *Alpha AMY* | 632 | *Alpha AMY* | 14.83 | *GBE1* | 24 | *otsB* | 1.55 | *PYG* | 9.105 | *PYG* | 3.89 |
| 5 | *Alpha AMY* | 288 | *Alpha AMY* | 12 | *SUS* | 19.65 | *SUS* | 592 | *INV* | 14.50 | *PYG* | 24 | *TPS8* | 1.52 | *GBE1* | 8.973 | *GBE1* | 3.89 |
| 6 | *SUS* | 240 | *PGM* | 11 | *GBE1* | 15.96 | *GBE1* | 544 | *PGM* | 14.33 | *PGM* | 22 | *PGM* | 1.49 | *PGM* | 8.914 | *PGM* | 3.83 |
| 7 | *glgA* | 240 | *glgC* | 9 | *PYG* | 15.96 | *PYG* | 544 | *otsB* | 14.33 | *INV* | 22 | *TPS6* | 1.43 | *INV* | 8.418 | *otsB* | 3.83 |
| 8 | *glgC* | 240 | *HK* | 9 | *PGM* | 10.17 | *PGM* | 344 | *HK* | 13.33 | *glgC* | 18 | *GBE1* | 1.40 | *glgC* | 8.316 | *HK* | 3.72 |
| 9 | *Beta AMY* | 48 | *scrK* | 8 | *glgC* | 8.60 | *HK* | 168 | *scrK* | 12.83 | *HK* | 18 | *PYG* | 1.40 | *HK* | 8.239 | *scrK* | 3.67 |
| 10 | *HK* | 24 | *ISA* | 8 | *HK* | 4.52 | *glgC* | 152 | *glgC* | 12.75 | *scrK* | 16 | *SUS* | 1.35 | *scrK* | 8.176 | *glgC* | 3.50 |

Table C.3 The calculation result of genes related to glycolysis pathway by Cytohubba

| Rank | MCC | | MNC | | Betweenness | | Stress | | Closeness | | Degree | | DMNC | | EPC | | Radiality | |
| --- | --- | --- | --- | --- | --- | --- | --- | --- | --- | --- | --- | --- | --- | --- | --- | --- | --- | --- |
|  | Name | Score | Name | Score | Name | Score | Name | Score | Name | Score | Name | Score | Name | Score | Name | Score | Name | Score |
| 1 | *TPI* | 976 | *TPI* | 12 | *TPI* | 35.60 | *ALDH* | 480 | *TPI* | 13.50 | *TPI* | 24 | *PGM* | 1.34 | *TPI* | 6.892 | *TPI* | 3.07 |
| 2 | *PGM* | 960 | *PK* | 10 | *ALDH* | 29.19 | *TPI* | 456 | *LDH* | 12.50 | *PK* | 20 | *HK* | 1.28 | *LDH* | 6.715 | *LDH* | 2.93 |
| 3 | *PK* | 898 | *LDH* | 10 | *E5.1.3.15* | 20.75 | *E5.1.3.15* | 272 | *PK* | 12.33 | *LDH* | 20 | *PFP* | 1.24 | *PK* | 6.559 | *PK* | 2.87 |
| 4 | *LDH* | 858 | *PGM* | 9 | *LDH* | 15.52 | *LDH* | 264 | *E5.1.3.15* | 12.00 | *PGM* | 18 | *NAPH+* | 1.22 | *PGM* | 6.332 | *E5.1.3.15* | 2.87 |
| 5 | *PFKA* | 842 | *E5.1.3.15* | 9 | *PK* | 12.65 | *PK* | 160 | *PGM* | 11.83 | *E5.1.3.15* | 18 | *PFKA* | 1.17 | *E5.1.3.15* | 6.299 | *PGM* | 2.80 |
| 6 | *HK* | 792 | *ALDO* | 8 | *ACSS1_2* | 9.62 | *ACSS1_2* | 144 | *NAPH+* | 11.50 | *ALDO* | 16 | *PK* | 1.08 | *PFKA* | 6.283 | *NAPH+* | 2.80 |
| 7 | *NAPH+* | 756 | *HK* | 8 | *ALDO* | 6.09 | *NAPH+* | 120 | *ALDO* | 11.33 | *HK* | 16 | *ALDO* | 1.05 | *HK* | 6.226 | *PFKA* | 2.73 |
| 8 | *ALDO* | 174 | *NAPH+* | 8 | *NAPH+* | 5.29 | *ALDO* | 112 | *HK* | 11.33 | *NAPH+* | 16 | *LDH* | 1.04 | *NAPH+* | 6.191 | *HK* | 2.73 |
| 9 | *PFP* | 96 | *PFKA* | 8 | *PFKA* | 3.85 | *PGM* | 64 | *PFKA* | 11.33 | *PFKA* | 16 | *TPI* | 0.97 | *ALDO* | 6.053 | *ALDO* | 2.73 |
| 10 | *E5.1.3.15* | 82 | *ACSS1_2* | 6 | *PGM* | 2.77 | *PFKA* | 64 | *ALDH* | 10.50 | *ALDH* | 12 | *ALDH* | 0.91 | *PFP* | 5.381 | *ALDH* | 2.67 |

**Appendix D.** **Primer sequences**

Table D.1 Primer design results

| ID | Name | F/R | Primer sequence | Size | Tm | GC% |
| --- | --- | --- | --- | --- | --- | --- |
| *AUR62026335* | *NAPH+_4* | F | AAGCCGTCAGTTTGCCAGAT | 128 | 60.25 | 50 |
|  |  | R | TCTCGTCCTCCGTCTTGTGA | 128 | 60.25 | 55 |
| *AUR62041432* | *ALDO_1* | F | TGTGCTTCCTTTCATTTGCTGT | 102 | 59.31 | 40.91 |
|  |  | R | CACCAAGCTGCTGCATAAACA | 102 | 59.73 | 47.62 |
| *AUR62021270* | *PGM* | F | TCATAATGCCGTCAAGCTACCA | 140 | 59.83 | 45.45 |
|  |  | R | AGTACCAAGGGCAGGAAGGA | 140 | 60.18 | 55 |
| *AUR62005566* | *gapA* | F | ACTACCAACTGCCTTGCTCC | 146 | 59.96 | 55 |
|  |  | R | GCTCTTCCACCCCTCCAATC | 146 | 60.11 | 60 |
| *AUR62025310* | *SUS_1* | F | CTTGCATAACAAGCGACCCT | 81 | 58.55 | 50 |
|  |  | R | TGTCTTAAAGAGAGTGGGTTCAT | 81 | 57.17 | 39.13 |
| *AUR62001699* | *PYG_2* | F | GACACATAGTTGTCGTCAATGGT | 128 | 59.01 | 43.48 |
|  |  | R | GCTCACTAGCTTCAAGCCGA | 128 | 60.11 | 55 |
| *AUR62031934* | *HK* | F | TGGAACGTCAGATGTGTGTGAT | 137 | 59.96 | 45.45 |
|  |  | R | AGGTAAACCCGAGCTCCCTT | 137 | 60.55 | 55 |
| *AUR62031441* | *malZ_2* | F | TGGCAAATCGCTCAATTCCA | 150 | 58.46 | 45 |
|  |  | R | TGTCCTTCCCAATTATATGCTCGT | 150 | 59.9 | 41.67 |
| *AUR62033840* | *EGLU_6* | F | ATGGGGATGAGCTCTTGTGG | 86 | 59.45 | 55 |
|  |  | R | CCTAGACAGTAGTACGTATTCCAA | 86 | 57.07 | 41.67 |
| *AUR62020044* | *PP2C_3* | F | TGGAGCTCGGTACATTTGTGT | 134 | 59.65 | 47.62 |
|  |  | R | CATCCCACAGCCCATCACTT | 134 | 60.03 | 55 |
| *AUR62001926* | *ZEP_2* | F | TGTTTGGGCATACAGACGCT | 133 | 59.96 | 50 |
|  |  | R | AAGTAGCCACGAGATAGAAGC | 133 | 57.28 | 47.62 |
| *AUR62001756* | *CYP707A* | F | AGGGCCCAATTCTGTATCCTC | 150 | 59.22 | 52.38 |
|  |  | R | CAGCCCAAAAACTGAAATCATCT | 150 | 57.86 | 39.13 |
| *AUR62021168* | *ABA2* | F | AGCATTGTAGCAGGTTGTGGT | 131 | 60.2 | 47.62 |
|  |  | R | ATCGCGTAGGGAGAAACAC | 131 | 57.26 | 52.63 |
| *AUR62021271* | *GA20ox* | F | GCGATGAGCAAGCTATCCCT | 139 | 59.97 | 55 |
|  |  | R | CCGGTCTTTGACATGGTGGA | 139 | 59.96 | 55 |
| *AUR62016415* | *GA3ox* | F | CGGCTCGTGTATCCGGTG | 134 | 60.28 | 66.67 |
|  |  | R | ATTGGGCCAAAGTTGACGAG | 134 | 58.75 | 50 |

**Appendix E. Correlation between physiological and biochemical indicators**

Table E.1 Correlation between physiological and biochemical indicators

|  | Fructose | Glucose | Sucrose | Maltose | Soluble protein | Total amylase | Soluble sugar | Starch | NCED | ZEP | AAO | ABA8’-H | GA20ox | GA3ox | GA2ox | Water absorption rate | Germination rate | ABA | GA_3_ |
| --- | --- | --- | --- | --- | --- | --- | --- | --- | --- | --- | --- | --- | --- | --- | --- | --- | --- | --- | --- |
| Fructose | 1.000 |  |  |  |  |  |  |  |  |  |  |  |  |  |  |  |  |  |  |
| Glucose | 0.086 | 1.000 |  |  |  |  |  |  |  |  |  |  |  |  |  |  |  |  |  |
| Sucrose | -0.314 | -.943** | 1.000 |  |  |  |  |  |  |  |  |  |  |  |  |  |  |  |  |
| Maltose | 0.200 | .829* | -0.771 | 1.000 |  |  |  |  |  |  |  |  |  |  |  |  |  |  |  |
| Soluble protein | 0.486 | 0.429 | -0.486 | 0.029 | 1.000 |  |  |  |  |  |  |  |  |  |  |  |  |  |  |
| Total amylase | -0.029 | -0.600 | 0.543 | -0.200 | -0.714 | 1.000 |  |  |  |  |  |  |  |  |  |  |  |  |  |
| Soluble sugar | 0.086 | 0.657 | -0.714 | 0.257 | 0.600 | -.886* | 1.000 |  |  |  |  |  |  |  |  |  |  |  |  |
| Starch | 0.143 | -.943** | .886* | -0.657 | -0.371 | 0.657 | -0.771 | 1.000 |  |  |  |  |  |  |  |  |  |  |  |
| NCED | 0.086 | .886* | -.943** | 0.600 | 0.429 | -0.600 | .829* | -.943** | 1.000 |  |  |  |  |  |  |  |  |  |  |
| ZEP | 0.086 | .886* | -.943** | 0.600 | 0.429 | -0.600 | .829* | -.943** | 1.000** | 1.000 |  |  |  |  |  |  |  |  |  |
| AAO | -0.257 | 0.600 | -0.657 | 0.429 | -0.086 | -0.314 | 0.657 | -0.771 | .829* | .829* | 1.000 |  |  |  |  |  |  |  |  |
| ABA8’-H | 0.086 | 0.657 | -0.771 | 0.429 | 0.257 | -0.600 | .886* | -0.771 | .886* | .886* | .886* | 1.000 |  |  |  |  |  |  |  |
| GA20ox | -0.257 | .829* | -0.771 | 0.429 | 0.429 | -0.600 | 0.714 | -.943** | .886* | .886* | 0.714 | 0.657 | 1.000 |  |  |  |  |  |  |
| GA3ox | 0.086 | 1.000** | -.943** | .829* | 0.429 | -0.600 | 0.657 | -.943** | .886* | .886* | 0.600 | 0.657 | .829* | 1.000 |  |  |  |  |  |
| GA2ox | 0.086 | .886* | -.943** | 0.600 | 0.429 | -0.600 | .829* | -.943** | 1.000** | 1.000** | .829* | .886* | .886* | .886* | 1.000 |  |  |  |  |
| Water absorption rate | -0.143 | .943** | -.886* | 0.657 | 0.371 | -0.657 | 0.771 | -1.000** | .943** | .943** | 0.771 | 0.771 | .943** | .943** | .943** | 1.000 |  |  |  |
| Germination rate | -0.100 | .900* | -0.800 | 0.400 | 0.500 | -1.000** | .900* | -1.000** | .900* | .900* | 0.600 | 0.700 | .900* | .900* | .900* | 1.000** | 1.000 |  |  |
| ABA | -0.143 | 0.429 | -0.486 | 0.600 | -0.486 | 0.086 | 0.257 | -0.486 | 0.543 | 0.543 | .829* | 0.657 | 0.314 | 0.429 | 0.543 | 0.486 | 0.100 | 1.000 |  |
| GA_3_ | -0.657 | 0.029 | 0.086 | 0.029 | -0.486 | 0.486 | -0.371 | -0.143 | 0.029 | 0.029 | 0.257 | -0.200 | 0.314 | 0.029 | 0.029 | 0.143 | -0.500 | 0.257 | 1.000 |
